# Direct Analysis of Marine Dissolved Organic Matter Using LC-FT-ICR MS

**DOI:** 10.1021/acs.est.3c07219

**Published:** 2024-03-01

**Authors:** Oliver J. Lechtenfeld, Jan Kaesler, Elaine K. Jennings, Boris P. Koch

**Affiliations:** † Department of Environmental Analytical Chemistry, Research Group BioGeoOmics, Helmholtz Centre for Environmental Research − UFZ, Permoserstraße 15, 04318 Leipzig, Germany; ‡ ProVIS−Centre for Chemical Microscopy, Helmholtz Centre for Environmental Research − UFZ, Permoserstraße 15, 04318 Leipzig, Germany; § Alfred-Wegener-Institut Helmholtz-Zentrum für Polar- und Meeresforschung, Am Handelshafen 12, 27570 Bremerhaven, Germany; ∥ University of Applied Sciences, An der Karlstadt 8, 27568 Bremerhaven, Germany

**Keywords:** Natural organic matter, Salt water, RP-LC-MS, Fourier transform ion cyclotron resonance mass spectrometry, PPL, SPE

## Abstract

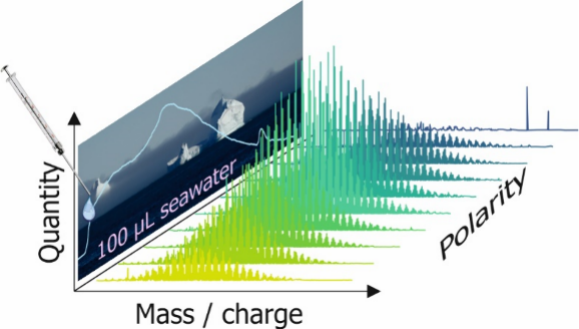

Marine dissolved organic matter (DOM) is an important component of the global carbon cycle, yet its intricate composition and the sea salt matrix pose major challenges for chemical analysis. We introduce a direct injection, reversed-phase liquid chromatography ultrahigh resolution mass spectrometry approach to analyze marine DOM without the need for solid-phase extraction. Effective separation of salt and DOM is achieved with a large chromatographic column and an extended isocratic aqueous step. Postcolumn dilution of the sample flow with buffer-free solvents and implementing a counter gradient reduced salt buildup in the ion source and resulted in excellent repeatability. With this method, over 5,500 unique molecular formulas were detected from just 5.5 nmol carbon in 100 μL of filtered Arctic Ocean seawater. We observed a highly linear detector response for variable sample carbon concentrations and a high robustness against the salt matrix. Compared to solid-phase extracted DOM, our direct injection method demonstrated superior sensitivity for heteroatom-containing DOM. The direct analysis of seawater offers fast and simple sample preparation and avoids fractionation introduced by extraction. The method facilitates studies in environments, where only minimal sample volume is available e.g. in marine sediment pore water, ice cores, or permafrost soil solution. The small volume requirement also supports higher spatial (e.g., in soils) or temporal sample resolution (e.g., in culture experiments). Chromatographic separation adds further chemical information to molecular formulas, enhancing our understanding of marine biogeochemistry, chemodiversity, and ecological processes.

## Abstract 2

This article describes how effective salt separation revolutionizes DOM analysis in original seawater and improves accurate characterization of marine biogeochemistry.

## Introduction

Marine dissolved organic matter (DOM) constitutes a large active pool in the global carbon cycle (662 petagrams of carbon).^1^ DOM is among the most complex chemical mixtures on our planet posing the greatest challenges to even the most advanced analytical methods targeting comprehensive chemical characterization and structural elucidation. Due to its unrivaled sensitivity and molecular resolution, ultrahigh resolution mass spectrometry has greatly advanced our understanding of the chemical composition and complexity of DOM. Each individual measurement reveals thousands of mass peaks, for which molecular formulas (MFs) can be assigned and, together with their respective intensities, are commonly exploited for DOM source and process studies.^2−5^

Analyzing marine DOM by mass spectrometry is impaired by the fact that the concentration of salts in the ocean exceeds the concentration of DOM by a factor of ∼35,000. Inorganic ions carry most of the charge in electrospray ionization (ESI), thus suppressing ionization of organic molecules. Consequently, the quantitative direct analysis of saltwater samples with ESI mass spectrometry has not been possible until now. DOM concentrations (measured as the proportion of dissolved organic carbon; DOC) of 40 μmol DOC L^–1^ and below^1^ pose an additional challenge in terms of instrument sensitivity.

Previous approaches of separating the sea salt and enriching the marine DOM to improve analytical sensitivity and robustness often used solid-phase extraction (SPE). However, SPE using a state-of-the art divinylbenzene-based polymer (PPL) only yields approximately 40–60% of the DOC from seawater,^6,7^ and the chemical composition of the extracts is strongly influenced by the chemistry of the adsorbant,^8,9^ the loading of the column,^8,10^ and the volume and type of eluent.^11^ In study areas with highly variable contributions of different DOM sources (e.g., estuaries, phytoplankton blooms),^12,13^ matrix-effects during solid-phase extraction represent an additional challenge for data interpretation.^14,15^

Liquid chromatography-mass spectrometry (LC-MS) using ultrahigh resolution mass spectrometers is increasingly applied to natural organic matter and petroleum samples to improve the physicochemical understanding by determining polarity and size distribution.^16−22^ However, major questions remain with regard to the representativeness of extracts.^23−25^ Due to the variable and largely unknown extraction efficiency of individual DOM compounds, it is difficult if not impossible, to quantitatively relate the molecular composition of extracts to the composition of DOM in original samples hampering our understanding of marine biogeochemical cycles.

From these challenges, several key requirements emerge for improving DOM analysis in seawater:

(i) Simplifying marine DOM sample preparation by removing the need for sample extraction.
(ii) Improving the representativeness of marine DOM composition as seen by FT-ICR MS.
(iii) Improving comparability of the analytical results with other bulk and molecular-level chemical techniques.
(iv) Leveraging the quantitative potential of nontargeted mass spectrometric analysis of complex DOM.

Such advancements would require to analyze non-extracted seawater samples - a method that is not yet available.

Here we present a new method for DOM characterization, which allows direct injection of original (i.e., non-extracted), filtered ocean water samples at native salt and DOC concentrations. To achieve this, we used reversed-phase liquid chromatography, enhanced by an isocratic elution step and a postcolumn counter gradient, hyphenated with Fourier transform ion cyclotron resonance mass spectrometry (LC-FT-ICR MS). The method is tested with DOM samples of varying carbon and salt concentrations from the Central Arctic Ocean and peat pore water. We evaluated the linearity of mass peak magnitudes for variable DOC concentrations, repeatability and intermediate precision, robustness against salt matrix and changing sample pH, and compared the results with traditional analysis of seawater DOM SPE extracts.

## Methods

### Samples and Chemicals

Seawater samples AO_low_ (55 μmol DOC L^–1^) and AO_high_ (88 μmol DOC L^–1^) were collected in surface water of the central Arctic Ocean during RV *Polarstern* cruise PS122/3 in spring 2020 (Table SI 1). One liter of seawater was filtered through precombusted glass fiber filters (500 °C, 5 h, Whatman, GF/F, approximately 0.7 μm nominal pore size), and aliquotes of the samples were immediately frozen at −20 °C until analysis. Original seawater samples were thawed and filtered again immediately before analysis (0.2 μm, Minisart RC4, Sartorius, Goettingen, Germany) to remove any particles that may have formed after melting the samples.

500 mL of the filtered sample AO_high_ was acidified to pH 3 (HCl, ultrapure; Merck) and extracted on board using a standard method^6^ (Bond Elut PPL, 200 mg, Agilent). DOM was eluted with 2 mL of MeOH (HPLC grade, Merck) and stored at −20 °C to minimize esterification.^26^ The carbon-based extraction efficiency was ∼40%, comparable to other marine waters.^7,27^ The extract was diluted (via evaporation of MeOH and reconstitution in ultrapure water using ultrasonication) to the same concentration as the original sample (AO_high_^SPE^: 88 μmol DOC L^–1^) immediately prior LC injections.

A peat pore water (PPW) sample was collected from the Neustädter Moor (Lower Saxony, Germany).^28^ The sample was diluted with ultrapure water (Milli-Q Integral 5, Merck, Darmstadt, Germany) to match marine DOC concentrations (PPW^S0^; 20–160 μmol DOC L^–1^). NaCl (p.a. Merck, baked at 400 °C for 4 h) was added to simulate salt concentrations of a seawater (salinity: 35; PPW^S35^), an estuarine (salinity: 17; PPW^S17^), and a sea ice brine sample (salinity: 70; PPW^S70^). These salt amended PPW-DOM samples were used for method validation (for an overview and complete list of samples used in this study, cf. Table SI 2, Figure SI 1).

For instrument quality control, 10 mg L^–1^ Suwannee River Fulvic Acid standard (SRFA, 2S101H, International Humic Substances Society) spiked with a set of previously used model compounds (Table SI 3) was used.^23,28^

### Reversed-Phase Liquid Chromatography

A recent reversed-phased liquid chromatography (RPLC) method for the direct injection of water samples^25,28^ was modified to account for the very high salt concentration in seawater. The system consisted of an ultrahigh pressure chromatography system (UltiMate 3000RS, Thermo Fischer Scientific, Waltham, U.S.A.), equipped with a binary high-pressure pump (HPG-3200RS), an auxiliary quaternary low pressure gradient pump (LPG-3400SD), an autosampler (WPS-3000TRS), a column oven (TCC-3000RS), and a diode array detector (DAD-3000RS). DOM separation was performed with a polar end-capped C-18 reversed-phase column (ACQUITY HSS T3, 1.8 μm, 100 Å, 150 × 3 mm, Waters, Milford, U.S.A.) equipped with a guard column (ACQUITY UPLC HSS T3 VanGuard, 100 Å, 1.8 μm, 2.1 mm × 5 mm), at a column temperature of 30 °C. As mobile phases, ultrapure water (adjusted with 0.1% formic acid and ammonium hydroxide (NH_4_OH) to reach pH 3) and methanol (LC-MS-grade, Biosolve, Valkenswaard, Netherlands, with same amounts of formic acid and NH_4_OH) were used. The flow rate was set to 0.2 mL min^–1^. The same mobile phases and flow rate were used for the auxiliary pump but without modifiers. A mobile phase gradient (isocratic step with 100% ultrapure water for 3.5 min and linear increase to 100% MeOH within 14 min, then hold 100% MeOH for 9 min) was used. The auxiliary pump mirrored the gradient of the main pump with an additional delay of 4.5 min to account for the flow path differences between both pumps and the T-piece, which was installed after the LC column.

An adjustable flow splitter (QuickSplit #600-PO10–04, ASI, Richmond, CA, U.S.A.) was installed after the T-piece, and the combined flow (0.4 mL min^–1^) was divided between the DAD (0.3 mL min^–1^) and the MS (0.1 mL min^–1^). The time difference between both detectors was approximately 0.5 min. Immediately before the MS, a 2-way-6-port valve was programmed to divert the flow to waste during the time when most of the salt eluted from the column (switch to ESI at 10.5 min). The void volume of the system was approximately 0.9 mL (4.3 min), and the methanol from the gradient of the main pump first reached the MS after approximately 13 min. For the salt-free PPW and the PPL-extracted seawater samples, the 2-way-6-port valve was already switched at 5.5 min to record MS spectra for the early eluting DOM compounds (Figure SI 2).

An injection volume of 100 μL was used for all samples. The effect of sample pH on the separation was tested by adjusting sample AO_low_ to pH 3 with formic acid (AO_low_^pH3^, Table SI 2, Figure SI 1). With respect to carbon concentration, the seawater samples were not adjusted prior to injection in order to ensure a consistent matrix across samples.

### ESI-FT-ICR Mass Spectrometry

Mass spectra were obtained with an FT-ICR mass spectrometer equipped with a dynamically harmonized analyzer cell (solariX XR, Bruker Daltonics, Billerica, U.S.A.) and a 12 T refrigerated, actively shielded superconducting magnet (Bruker Biospin, Wissembourg, France). The data were acquired in negative ion mode with an ESI source (Apollo II, Bruker Daltonics, Billerica, U.S.A., capillary voltage: 4.3 kV) in full profile magnitude mode with a transient size of 4 MWord (∼1.6 s free induction decay, FID). The ion accumulation time (IAT) was set to 1.6 s, and the mass range was set to *m*/*z* 147–1000. The mass resolving power (m/Δm, full width half-maximum) at *m*/*z* 400 was approximately 500,000 ± 40,000, which is sufficient to resolve all major DOM ions in the considered mass range. SRFA spiked with model compounds was measured with 0.5 s IAT (cf. SI Text: Instrument Quality Control).

As reference to state-of-the art analysis, the PPL extracted seawater sample (AO_high_^SPE^) was diluted to 0.8 mmol DOC L^–1^ (10 mg DOC L^–1^) in ultrapure water and MeOH (50/50, v/v) and measured with the standard direct infusion (DI-) FT-ICR MS method (256 scans, 4 MWord, 8 ms IAT, ESI(−), 4 μL min^–1^).

### Data Processing

#### Segmentation of LC-FT-ICR MS Spectra

**Figure 1:**
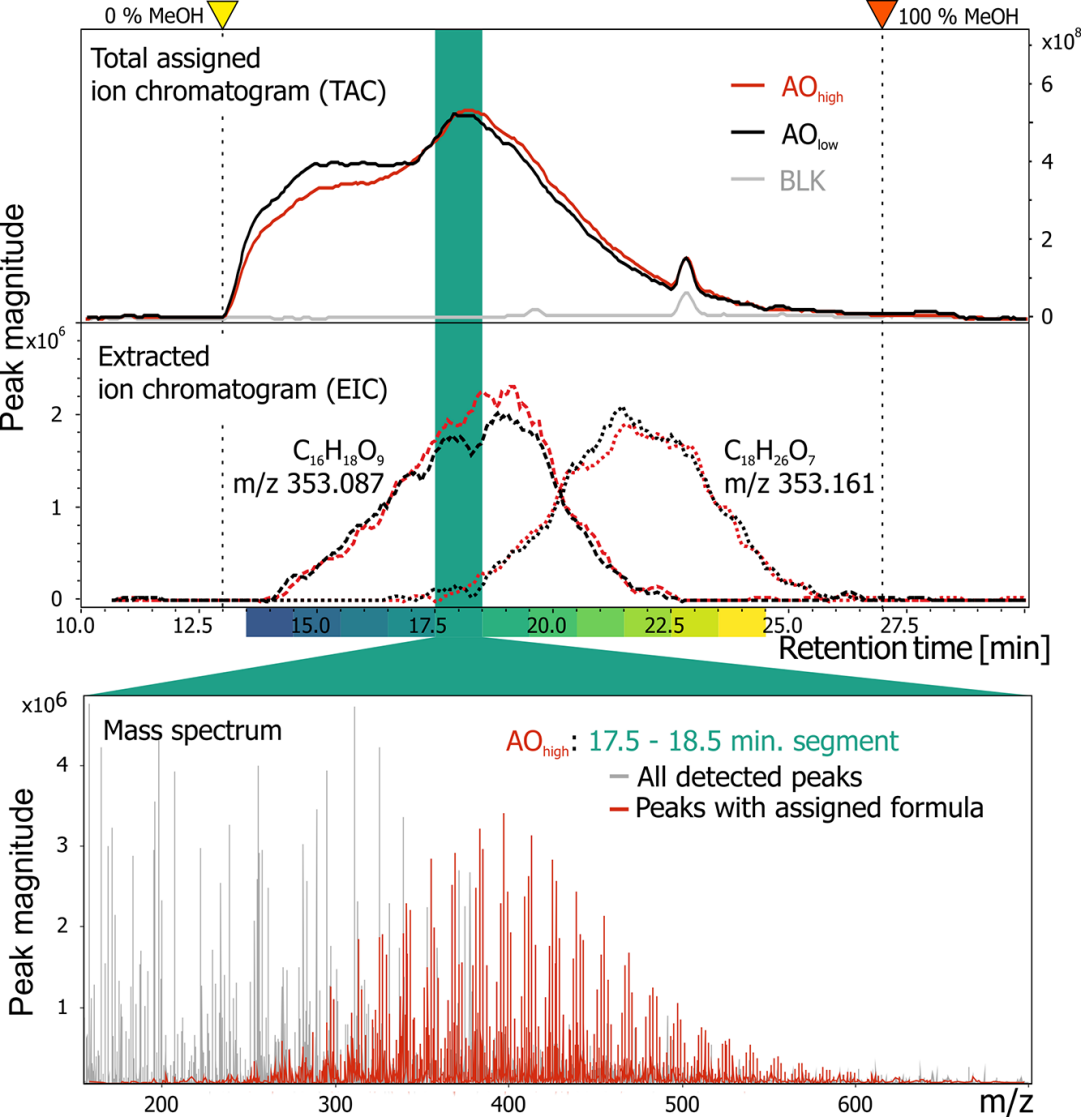
Total assigned ion chromatograms (TAC, solid lines, right *y*-axis) and extracted ion chromatograms (EIC, dashed lines, left *y*-axis) for two Arctic Ocean samples (AO_high_ (red) and AO_low_ (black)) and a pure water blank injection (BLK, gray). Two *m*/*z* values on the same nominal mass were selected, representing molecular formulas with higher O/C (*m*/*z* 353.0878: C_16_H_18_O_9_) and lower O/C ratios (*m*/*z* 353.1606: C_18_H_26_O_7_). The yellow marker and dashed line indicate the retention time at which the first methanol reaches the MS (13 min). 100% methanol was reached at 27 min (red marker). The color bars indicate the LC retention time segments, highlighted in subsequent figures. TAC represents 35% of the TIC in this example.

Seawater DOM elution profiles in the retention time range from 13 to 25 min did not show distinguishable chromatographic features (Figure 1). LC-FT-ICR MS-derived single scan full profile spectra were therefore binned into 1 min segments using a custom script in DataAnalysis (Version 6, Bruker, cf. SI Script for DataAnalysis; start: 13.5 min, end: 24.5 min), resulting in 11 segments. In the last two segments (22.5–24.5 min) only the PPL extracted sample showed typical DOM spectra, and these segments were not evaluated for the original seawater samples. For the tests with PPW and the PPL-extracted seawater samples, seven segments for the early eluting DOM (6.5–13.5 min) were additionally included due to the earlier valve switching. For the seawater samples, these segments were not available due to the valve setting, directing the flow to waste during the initial elution of salt (Figure SI 2). Segment-wise retention times (RT) are reported using the mean RT of the respective segment (e.g., 14 min for the segment 13.5–14.5 min).

#### Calibration and Molecular Formula Assignment

Averaged LC-FT-ICR MS and DI spectra were internally recalibrated with a list of known DOM masses (*n* = 425; 150 < *m*/*z* < 980), resulting in an average root-mean-squared error of 0.15 ppm across segments and samples (*n* = 165). Molecular formulas (MFs) were assigned in the mass range 150–1000 Da with a maximum tolerated mass error of ±0.5 ppm and element ranges C:1–60, H:1–122, O:0–40, N:0–2, S:0–1 using an in-house software. We also considered Na for samples PPW^S0^ and PPW^S35^. Tentative Na-adducts ([M – 2H^+^ + Na^+^]^−^) for highly polar DOM molecules were identified by linking a molecular formula (C_c_H_h_N_n_O_o_S_s_) to its potential Na-adduct (C_c_H_h-1_Na_1_N_n_O_o_S_s_). To distinguish between MFs assigned to DI-FT-ICR MS and LC-FT-ICR MS data, we refer to molecular features as molecular formulas detected at a given retention time (here: in a distinct segment).

#### Blank Correction

Pure water injections were measured in triplicates across the sample sequence and processed in the same way as the samples. The MFs in the individual blank segments were subtracted from the list of MFs of the respective segments of the sample. Subtraction was based on the presence of a molecular formula in any of the three pure water injections. A full method blank was not included, but we additionally excluded MFs commonly found as contaminants in DOM samples^29^ (cf. SI List of Surfactants). For the DI measurement, an instrument blank was subtracted accordingly.

#### Data Visualization

To visualize chromatographic performance, total ion chromatograms (TIC, summed magnitude of all detected mass peaks in each mass spectrum), total assigned ion chromatograms (TAC, summed magnitude of all mass peaks with molecular formula assignment in each mass spectrum),^19^ and extracted ion chromatograms (EIC) were used. Chromatogram data were smoothed by Savitzky-Golay with 4 (TIC, TAC) and 11 (EIC) points and 1 cycle in DataAnalysis. Individual MFs and aggregated molecular descriptors (e.g., intensity weighted average H/C and O/C ratios) were plotted in the chemical H/C-vs-O/C or H/C-vs-mass space to visualize compositional differences.^30^ Molecular formula based biogeochemical indices reported for marine DOM (I_DEG_,^31^ IOS,^4^ I_Terr_, t-Peaks^32^) were calculated, and the EICs were extracted from LC-FT-ICR MS chromatograms.

For an overview of the data processing pipeline used in this study, refer to Figure SI 3.

### Method Assessment

We assessed the performance and robustness of the method according to the following criteria:

(i) Linear detector response and sensitivity: PPW was injected at different concentrations (20, 40, 80, 160 μmol DOC L^–1^) covering the typical seawater DOC concentration range (PPW^S0^ samples) and measured with LC-FT-ICR MS. The number of assigned MFs and the total assigned intensity (i.e., sum of intensity of all peaks having a formula assignment) and a linear regression between DOC concentration and total/individual MFs intensity was used to evaluate the detector sensitivity and linearity, respectively.
(ii) Robustness: The effect of salt on the DOM mass spectra from LC-FT-ICR MS was assessed in two ways: First, 35 g L^–1^ NaCl was added to the PPW samples (PPW^S35^ samples) prepared at different concentrations (40, 80, 160 μmol DOC L^–1^) to evaluate the potential masking of polar DOM molecules due to coelution of salt and compared the detector response of original and salt amended PPW. Second, we checked for potential adducts from residual salt in the PPW^S35^ sample and compared it to PPW^S0^. Finally, the effect of sample pH on the retention of polar DOM was tested by adding formic acid to sample AO_low_.
(iii) Repeatability and intermediate precision: Repeatability was determined by measuring the sample (AO_low_) in triplicate with LC-FT-ICR MS, and the number of shared MFs and the coefficient of variation (CV) of raw mass peak magnitudes (hereafter: peak intensities) were evaluated. Intermediate precision was assessed using the CV of peak areas of model compounds for 11 injections during a multiday measurement.
(iv) Comparison with PPL extracts. The PPL extracted sample (AO_high_^SPE^) was measured with LC-FT-ICR MS (diluted to the same concentration as the original sample, 88 μmol DOC L^–1^) and DI-FT-ICR MS (diluted to 10 mg DOC L^–1^/0.8 mmol DOC L^–1^) and compared to the original sample (AO_high_) measured with LC-FT-ICR MS (Table SI 2). The relative difference of the peak intensities was evaluated to test the effect of extraction on the observable molecular composition. Further, an intensity averaged pseudo-DI measurement was calculated from the LC-FT-ICR MS segments. MFs solely detected in one sample and those shared between the three samples were evaluated based on their number and molecular descriptors.

More details about the validation steps can be found in SI Text: Method Assessment and Table SI 2.

## Results and Discussion

### Chemodiversity and Polarity of Marine DOM from Original Seawater Samples

Our LC-FT-ICR MS method allowed the direct injection of 100 μL of filtered seawater resulting in more than 200 single DOM mass spectra at a mass resolving power of ∼500,000 at *m*/*z* 400. The marine DOM eluted from the column in a broad, unstructured peak between 13 and 25 min (Figure 1), as observed previously for aquatic DOM extracts.^23,28,33−35^ Owing to the very low amount of injected DOM and long accumulation times (here: 1.6 s), the total ion chromatogram (TIC) showed a less pronounced DOM peak as compared to previous studies using concentrated DOM extracts.^23,36^ However, DOM sample TICs were clearly distinguishable from blank injections (Figure SI 4), and the total assigned ion chromatograms (TAC) clearly showed the DOM elution profile (Figure 1). Compared to a peak width of individual model compounds of less than 0.5 min (full-width half-maximum (FWHM), cf. Table SI 3), peak widths of DOM *m*/*z* ratios were much wider (4–5 min FWHM, Figure 1), reflecting the large structural diversity of DOM.^35^ At the level of individual *m*/*z* ratios, the retention and separation of DOM indicated that the method is suitable for low concentrated seawater samples at native concentration as well as for extracted seawater or freshwater samples (Figure SI 5). However, the low concentration of DOM in ocean water required a longer IAT to collect enough ions for detection in the ICR cell. The FT-ICR MS transient length was also extended (4 MWord, ∼1.6 s with start at *m*/*z* 147) to maximize sensitivity, resolution, and MS duty cycle. The resulting loss of time resolution as compared to our previous method^28^ using 2 MWord transients on the same 12 T FT-ICR instrument did not result in a substantial loss of chromatographic resolution (Tables SI 3,4, Figure SI 6).

**Figure 2:**
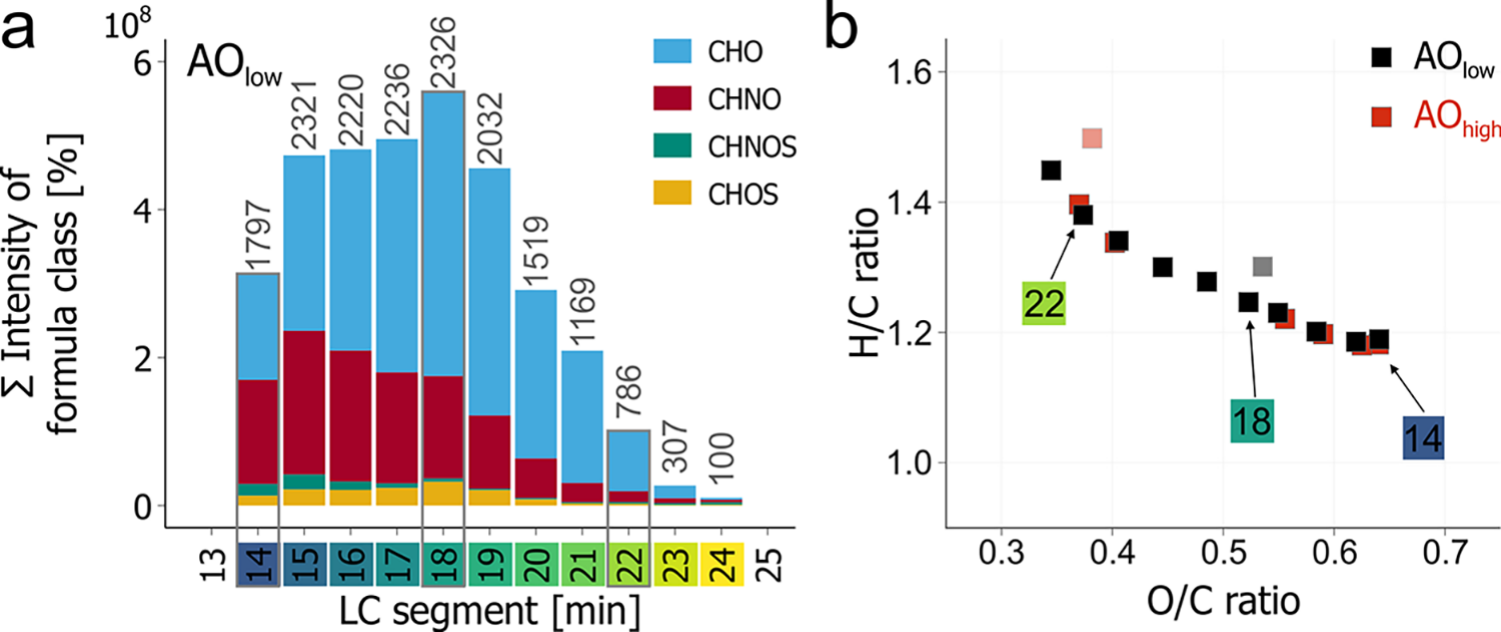
LC-FT-ICR MS analysis of original seawater samples. (a) Summed intensity of assigned molecular formulas (MFs) based on formula classes (colors in legend) for the AO_low_ sample over all 11 LC segments (14**–**24 min). Colors on the retention time axis relate to labels displayed in (b). (b) Intensity-weighted average molecular H/C and O/C ratios for all segments of two seawater samples measured at native concentration (AO_low_: 55 μmol DOC L^**–**1^, black and AO_high_: 88 μmol DOC L^**–**1^, red) with LC-FT-ICR MS. Note that the segment at 24 min (lighter colors) only contained very few MFs (AO_low_: 100, AO_high_: 174). For details for sample AO_low_, AO_high_ and AO_high_^SPE^, cf. Figures SI 8–11.

Both Arctic seawater samples resulted in a comparable summed intensity and number of detected molecular features (AO_high_: total features: 16,800, unique MFs: 5,800; AO_low_: total features: 16,250, unique MFs: 5,700) with the largest number of detected MFs eluting between 15 and 19 min (Figure 2a). H/C_wa_ increased and O/C_wa_ decreased with retention time (Figure SI 7), confirming the general connection between the mean O/C ratio and polarity (number of acidic groups) of marine DOM.^23,33^ Notably, all chromatographic segments showed a higher H/C_wa_ and lower O/C_wa_ ratio for seawater compared to peat pore water (Figure SI 7), which also is in agreement with previous DI^3^ and LC measurements^23^ of solid-phase extracted DOM. In addition, we found that the average molecular mass increased with retention time from *m*/*z* 400 at 14 min to *m*/*z* 478 at 24 min (Figures SI 7).

Direct injection and polarity separation of marine DOM also allow for novel insights into biogeochemical indices and marker MFs. Those MFs that constitute the degradation index^31^ (*I*_DEG_) eluted with the majority of the DOM (15–22 min, Figure SI 12) and the broad distribution are evidence that the formulas represent a multitude of structural isomers. Only a small shift in polarity could be observed between the *I*_DEG_-POS (MFs decreasing in intensity with radiocarbon age of marine DOM) and *I*_DEG_-NEG (MFs increasing in intensity) formulas, despite clear differences based on their molecular H/C ratios.^31^ In contrast, MFs from the terrestrial index (*I*_*Terr*_) showed a pronounced maximum of highly polar isomers related to the terrestrial markers (*Terr*; MFs with increased intensity in riverine DOM, Figure SI 13).^32^ This confirms previous observations (based on extracted samples) that terrestrial DOM can be distinguished by molecular descriptors and polarity.^23^ Although the isomeric overlap is still substantial, markers for marine (e.g., IOS-MFs)^4^ and terrestrial DOM (e.g., t-Peaks)^32^ cover distinct polarity regions in the LC chromatograms (Figure SI 14), providing new opportunities to resolve the compositional overlap of terrestrial and marine DOM.

### Enabling Direct Seawater DOM Analysis with LC-FT-ICR MS

Severe interferences due to the salt matrix have previously hampered the direct ESI-MS analysis of marine or other salt containing samples. We achieved an effective separation of salt from most of the DOM using a comparably large chromatographic column (providing a large pore volume) and an extended isocratic aqueous step after injection as compared to our previous work (Figure SI 2).^25,28^ Together, this resulted in a delayed DOM elution (main part of DOM elution > 13 min), while the salt passed the column with only little interaction with the stationary phase (Figure SI 15). Dilution of the sample with buffer free solvents via the auxiliary pump and a valve to direct the salt-containing flow to waste were important method adjustments to reduce salt-buildup inside the ESI source and on the cones, which otherwise limit the sensitivity and contribute to adduct formation during ionization. Overall, this resulted in a robust chromatography and stable mass peak intensities for long sample sequences (Tables SI 3,4; Figures SI 6,16). The counter gradient stabilized the solvent composition for ESI and reduced suppression effects from the buffered mobile phases of the primary pump.^28,37^ The counter gradient also allowed the postcolumn addition of an internal standard that can assist with lock-mass calibration and baseline drift correction.^38^

**Figure 3:**
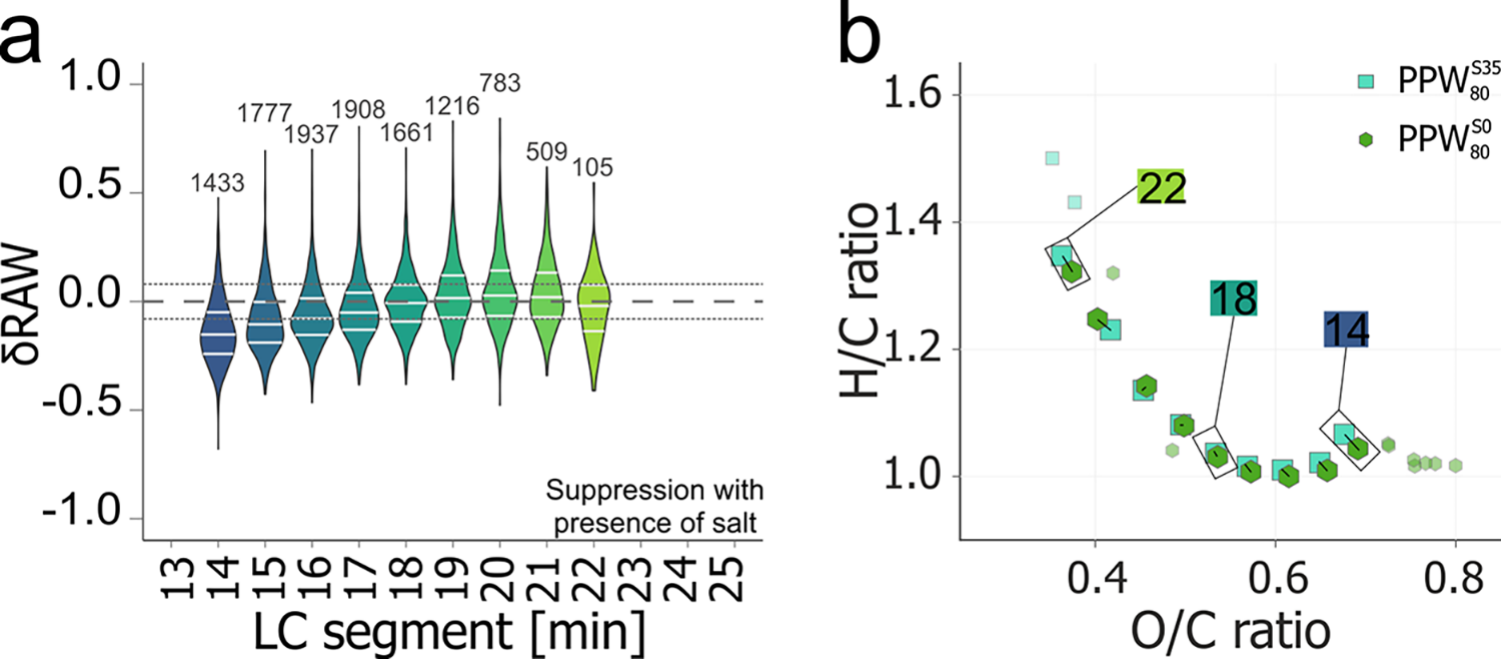
Effect of salt on the peak intensities of original peat pore water (PPW) measured by LC-FT-ICR MS. (a) Distribution of relative differences between peak intensities (δRAW) of molecular formulas (MFs) detected without (PPW^S0^) and with salt (PPW^S35^; 35 g L^–1^ NaCl) injected at 80 μmol DOC L^–1^ for individual LC-segments (14–22 min, # MFs: 105 < *n* < 1937; with 25th, median, and 75th percentile as white lines). The dashed lines indicate the peak intensity repeatability limits (cf. Figure SI 25). (b) Intensity-weighted average molecular H/C and O/C ratios for all segments between 14 and 22 min for PPW at 80 μmol DOC L^–1^ without (hexagons) and with salt (squares). Transparent symbols indicate segments < 14 min and > 22 min.

To demonstrate the suitability of the method for original seawater, we assessed which MFs were not accessible because they coeluted with the salt before 13 min and whether the salt affected segments after 13 min retention time. For this purpose, we added 35 g L^–1^ NaCl to PPW (i.e., PPW^S35^) having different concentrations (40, 80, 160 μmol DOC L^–1^) and compared the results with salt-free PPW^S0^ samples (Table SI 2, Figure SI 1). Expectedly, the presence of salt prevented the detection of the most polar DOM fraction (6–13 min; 18–20% of the total assigned intensity, Figures SI 17,18). However, the impact of salt was only small for DOM eluting at 14–16 min and negligible for retention times > 16 min (Figure SI 19). In the segments at 14–16 min, the salt primarily suppressed polar MFs (Figure 3a) resulting in a small shift to higher average H/C and lower O/C ratios, compared to the same concentration of salt-free PPW (Figure 3b). Although the majority of free salt eluted already in the column void volume (Figure SI 15, cf. SI Figure 10 in Jennings et al. (2022)), part of the oxygen-rich, polar DOM may have formed Na-adducts ([M – 2H^+^ + Na^+^]^−^) that were partially retained on the LC column (Figure SI 20). The Na-adducts usually remain undetected, if not explicitly accounted for during formula assignment for ESI(−) MS data. Notably, this effect was slightly higher for higher PPW concentrations (Figure SI 21).

### Analysis of Samples at Native DOM Concentrations

#### Linear Detector Response

**Figure 4:**
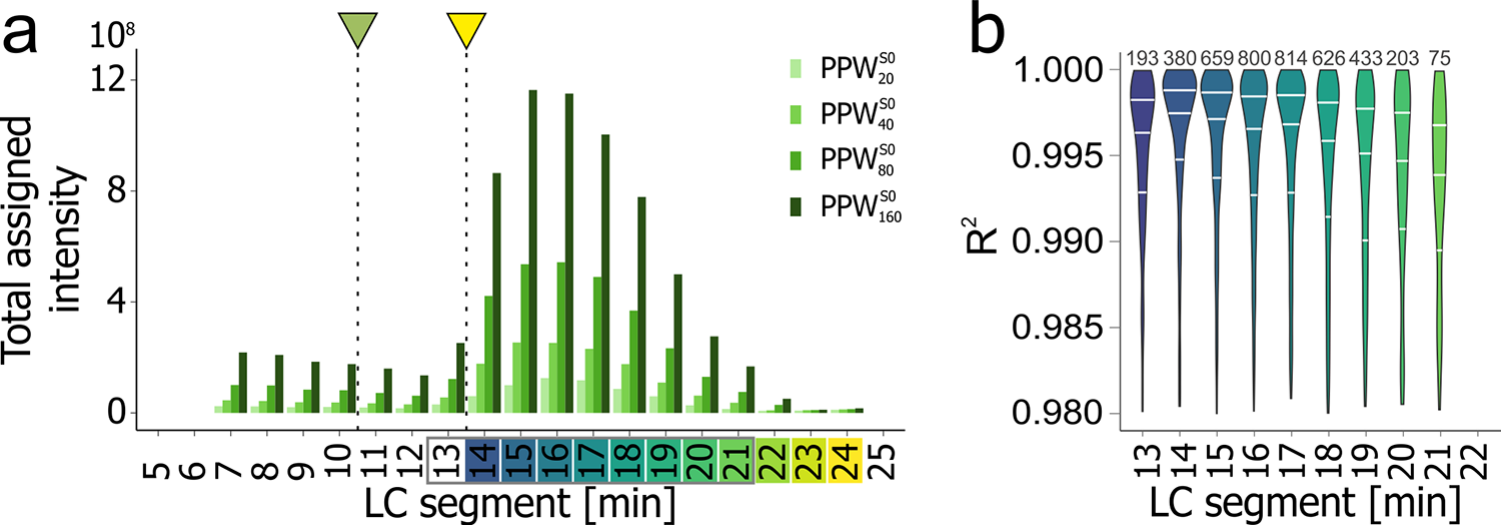
Linearity of detector response. (a) Summed intensity of assigned molecular formulas (MFs; total assigned intensity) in individual LC-segments (7–24 min, 18 segments) of different concentrations (20–160 μmol DOC L^–1^) of salt-free peat pore water (PPW^S0^). Without salt addition, DOM in early segments (<13 min) can be observed, which was otherwise masked by the coelution of the salt matrix (cf. Figure SI 23) and the respective time of valve switching (green marker). The yellow marker and dashed line indicate the retention time at which the first methanol reaches the MS (13 min). The gray box on the retention time axis indicates data displayed in (b). (b) Linear regression of peak intensities for MFs detected in segments of the PPW^S0^. MF with R^2^ values > 0.98 (corresponding to a significant linear regression at α = 0.01) are shown, representing 63% (21 min)–86% (16 min) of MFs that were detected at all concentrations (75 < *n* < 814; with 25th, median, and 75th percentile as white lines). Segments < 13 min and > 21 min were omitted due to the low number of detected MFs for the 20 μmol DOC L^–1^.

We tested how the peak intensities of MFs at a given retention time segment corresponded to the amount of DOC injected. For this purpose, salt-free PPW^S0^ was diluted to concentrations covering typical seawater DOC concentrations (20, 40, 80, and 160 μmol DOC L^–1^; equivalent to 2–16 nmol DOC injected, Figure SI 1). Based on MFs that were detected at all four concentration levels in a given retention time segment ≥ 14 min (# MFs: 75 < *n* < 814), 63% (at 21 min) to 86% (at 16 min) of the respective mass peak magnitudes showed a highly significant (*p* < 0.01) linear relationship with DOC concentration (Figure 4). Deviation from the linearity was related to peaks with lower magnitude or LC-derived contaminants (Figure SI 22).

Consequently, within each retention time segment, total assigned intensity (Figure 4a) and the number of MFs linearly increased with DOC concentration (Figure SI 17). Notably, a higher DOC concentration is likely to result in more MFs being detected, affecting the apparent molecular composition and requiring adjustments in data processing. However, if only shared MFs are considered, the aggregated molecular descriptors O/C_wa_ and H/C_wa_ of all segments in the PPW dilution series were highly similar, confirming that the molecular composition is largely conserved independent of sample concentration (Figure SI 17). Therefore, our method provides a sufficient linear range to accommodate the entire range of DOC concentrations expected for typical seawater samples.

Using the salt-amended PPW^S35^ samples, we found that the average molecular composition was well preserved across a concentration gradient (Figures SI 18,19), similar to the salt-free assessment. The detector response was highly linear and comparable to the test without salt (Figure SI 23). Expectedly, for the most polar segments, the sensitivity decreased (flatter slopes) reflecting the intensity suppression by salt adducts. Overall, this resulted in a lower detector response (based on total assigned intensity) of the saline samples (Figure SI 24). However, removing the first three segments that were most affected by the matrix (14–16 min), an almost identical response was observed for saline and salt-free PPW (Figure SI 24). Lower (17 g L^–1^) and higher (70 g L^–1^) salt concentrations still resulted in a linear detector response, indicating that a comparison of DOM samples from a salt gradient (e.g., estuary, sea ice) is possible.

The linear response of total assigned intensity with DOC concentration (Figure SI 24) was previously not achievable with SPE-based analyses and demonstrated the potential to semiquantitatively evaluate molecular features in DOM. However, absolute quantification can of course only be achieved in targeted molecular approaches for which standards are available.

#### Repeatability and Intermediate Precision

Sample AO_low_ was measured in triplicate to explore the mass peak magnitude variance as a function of magnitude^39^ and retention time. For all segments, the CVs of the peak intensities were below 10% for 60% of the MFs and below 18% for 90% of the MFs detected in the LC segments (380 < *n* < 1700, Figure SI 25). Similarly, between 51% (22 min) and 73% (18 min) of the MFs were detected in all three replicates, which in turn accounted for 71% to 95% of the total assigned intensity, similar to measurements of SRFA at much higher concentrations (Figure SI 26).^28^ As found for previous assessments, peak detection and repeatability were primarily dependent on peak magnitude.^28,40^ Using the LC-derived peak areas of the five model compounds spiked into SRFA, the method achieved 5–6% repeatability and 9–17% intermediate precision (Table SI 4).

#### Recommendations for Sample and Data Handling

An important advantage of our new method is that it drastically simplifies sample preparation and avoids the chemical fractionation that results from SPE. However, when working with very small volumes of saline water, it is very important to consider fractionation effects due to filtration. In our study, we still used a fairly large sample volume for filtration (1 L). When filtering minimal amounts of seawater (e.g., with syringe filters), it is important to consider that DOM can be absorbed on the filter surface.^41^ We therefore recommend cleaning and conditioning the filter with sufficient sample volume to avoid chemical fractionation. In DOM analysis, it is best to freeze the sample immediately after filtration.^42,43^ We do not recommend acidification with HCl, as it introduces additional inorganic ions, but acidification with formic acid to pH 3 and cooling might be an alternative option for sample storage. It is noteworthy that the peak intensity increased by 15 to 38% when samples were adjusted to pH 3 with formic acid prior to injection (Figures SI 27,28). Acidification leads to protonation of small, highly polar compounds, which can thus be better separated from the salt. The robustness of the method can be further improved for samples whose native pH values differ greatly (e.g., from an estuary) by adjusting the sample pH prior injection. We also recommend refiltration with 0.2 μm cellulose acetate filters to protect the LC from particles that may form during sample storage. Because of the highly linear relationship between sample DOC concentration and peak magnitude (Figure 4), we recommend measuring samples at their native concentration rather than adjusting DOC concentration prior to analysis, as is common with DI measurements.

Although the longer run time of LC-FT-ICR MS increases the cost of analysis compared to DI-FT-ICR MS measurements, it eliminates the time-consuming extraction step, saving time and chemicals during field campaigns.

### DOM Composition from Original Water versus SPE Extracts

#### Effect of PPL Extraction on the Observable DOM Chemodiversity

For all segments ≥ 14 min combined, the SPE extract of AO_high_ that was adjusted to 88 μmol DOC L^–1^ (AO_high_^SPE^) had a 59% higher total assigned intensity compared to the original AO_high_ sample at the same DOC concentration (data not shown). Also, the total number of molecular features differed, with 16,254 in the directly measured and 20,540 in the PPL extracted sample. Assuming that the bulk carbon extraction efficiency of 40% is reflected in a corresponding (average) loss of the TAC, the SPE extract in fact represents only 63% of the original seawater TAC, which is explained by the improved detection of well-ionizing polar compounds by direct LC-FT-ICR MS measurement (Figure SI 5).

**Figure 5:**
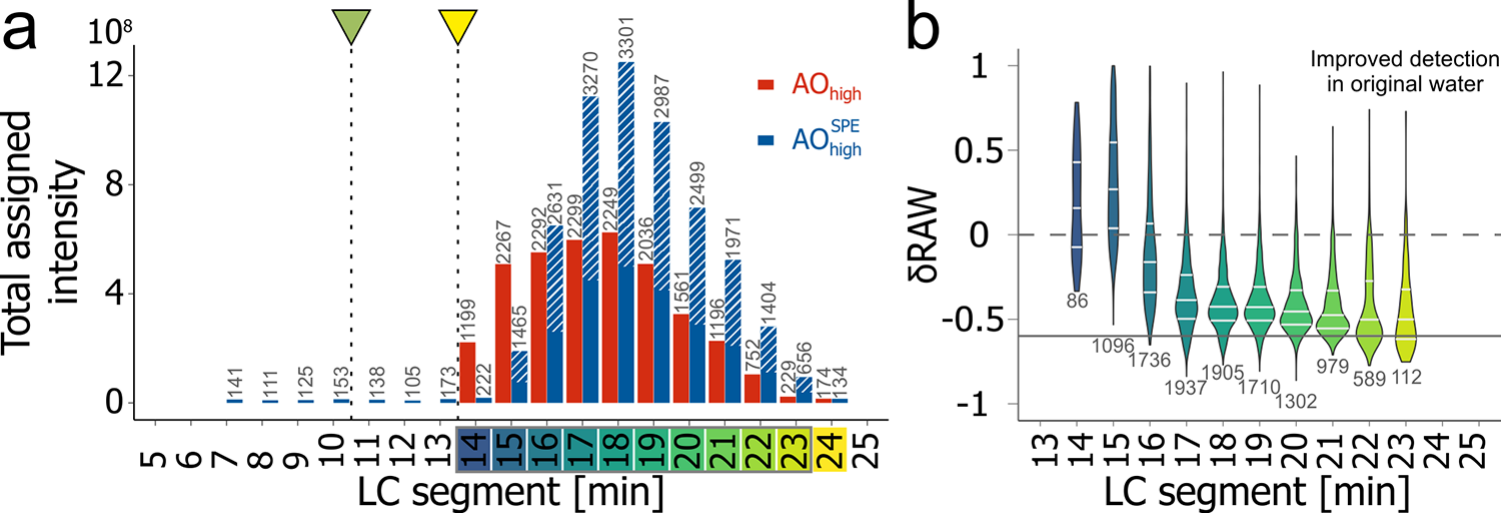
Comparison between an original (AO_high_, red) and PPL-extracted sample (AO_high_^SPE^, blue) measured at 88 μmol DOC L^–1^ with LC-FT-ICR MS. (a) Summed intensity of assigned molecular formulas (total assigned intensity) in each segment (7–24 min, 18 segments). No MFs were assigned to segments < 14 min in the original sample AO_high_ due to the coelution of salt and the respective time of valve switching (green marker). The yellow marker and dashed line indicate the retention time at which the first methanol reaches the MS (13 min). (b) Relative peak intensity difference (δRAW) of all shared MFs in original AO_high_ and PPL-extracted AO_high_^SPE^ seawater. Positive values indicate higher peak intensity in the original sample. The striped part of each bar for AO_high_^SPE^ in (a) and solid line in (b) indicate the mean loss in intensity according to the bulk carbon-based extraction efficiency (40%).

Accordingly, more polar segments contained a larger number of MFs and higher peak intensities compared to the extract (Figure 5, Figure SI 8). The early segments (7–13 min) were not accessible with the new method (coelution of salt) but accounted for only ∼1% of the summed intensity and 6% of the individual MFs of the extract (Figure SI 8). However, SPE with hydrophobic resins such as PPL also results in a loss of the most polar DOM compounds.^9,25,28^ The highly polar DOM fraction that could still be observed in the extract (<14 min) corresponded to 711 distinct MFs, of which one-third (*n* = 233) was also detected in the measurement of the original water sample (with RT ≥ 14 min). Early eluting unassigned mass peaks in the measurement of AO_high_^SPE^ probably represent chemical noise (Figure SI 29), possibly from silanol compounds derived from the chromatographic column. In contrast to the results obtained by adding salt to the PPW, the loss of intensity and number of unique MFs due to the coeluting salt in seawater samples as compared to their PPL extracts is much smaller and can be attributed to the unavoidable loss of such polar DOM compounds during the SPE process. The evaluation of specific biogeochemical markers for polar terrestrial DOM clearly demonstrated that SPE, even if combined with LC-FT-ICR MS, misses some of the most polar DOM compounds (Figures SI 13,14).

In the segments ≥ 14 min, 5,692 MFs were detected in the original sample (AO_high_), as compared to 6,607 MFs in its SPE extract AO_high_^SPE^. Out of those, 3,951 MFs were detected in both analyses (Figures SI 30,31). Although the majority of DOM in the SPE extract eluted at distinctly larger retention time as compared to the original sample (Figure 5, Figure SI 32), we observed a consistent shift toward lower H/C values considering each segment individually (Figure SI 7). This points to an additional extraction bias at the DOM isomer level, an effect previously not observable with DI methods. Notably, the shift in molecular composition due to the elimination of extraction bias will also impact ionization during ESI(−), and the observed relative peak intensity differences (δRAW, Figure 5b) thus only reflect the change in detector response. However, the spread in δRAW values indicated that extraction efficiencies for individual DOM may be studied in the future.

The overall higher intensity and number of detected MFs in the SPE extract were mainly driven by the later eluting, less polar DOM fractions and can be attributed to the selective enrichment of nonpolar compounds on the PPL sorbent.^9^ Our results agree with results from effluent samples,^25^ where also a negligible contribution of polar DOM and a proportionally higher contribution of less polar DOM were found in SPE extracts, when injected at the same DOC concentration. In the PPL-extracted AO_high_^SPE^, a higher fraction of CHO MFs (48%) was found as compared to the original sample (42%, Figure 5, Table SI 5). The comparable proportion of CHNO formulas (∼36%, Table SI 5) in the original and PPL extracted sample indicated that despite lower recovery of nitrogen-containing DOM by SPE these compounds profit from the reduced ionization suppression as compared to DI-FT-ICR MS.

We conclude that the loss of the most polar fractions due to coeluting salt is negligible compared to LC-FT-ICR MS of SPE extracts and that, in contrast, more polar compounds can be studied with the new direct injection LC-FT-ICR MS method.

#### Comparison of Conventional Direct Infusion and Original Water LC Analysis

**Figure 6:**
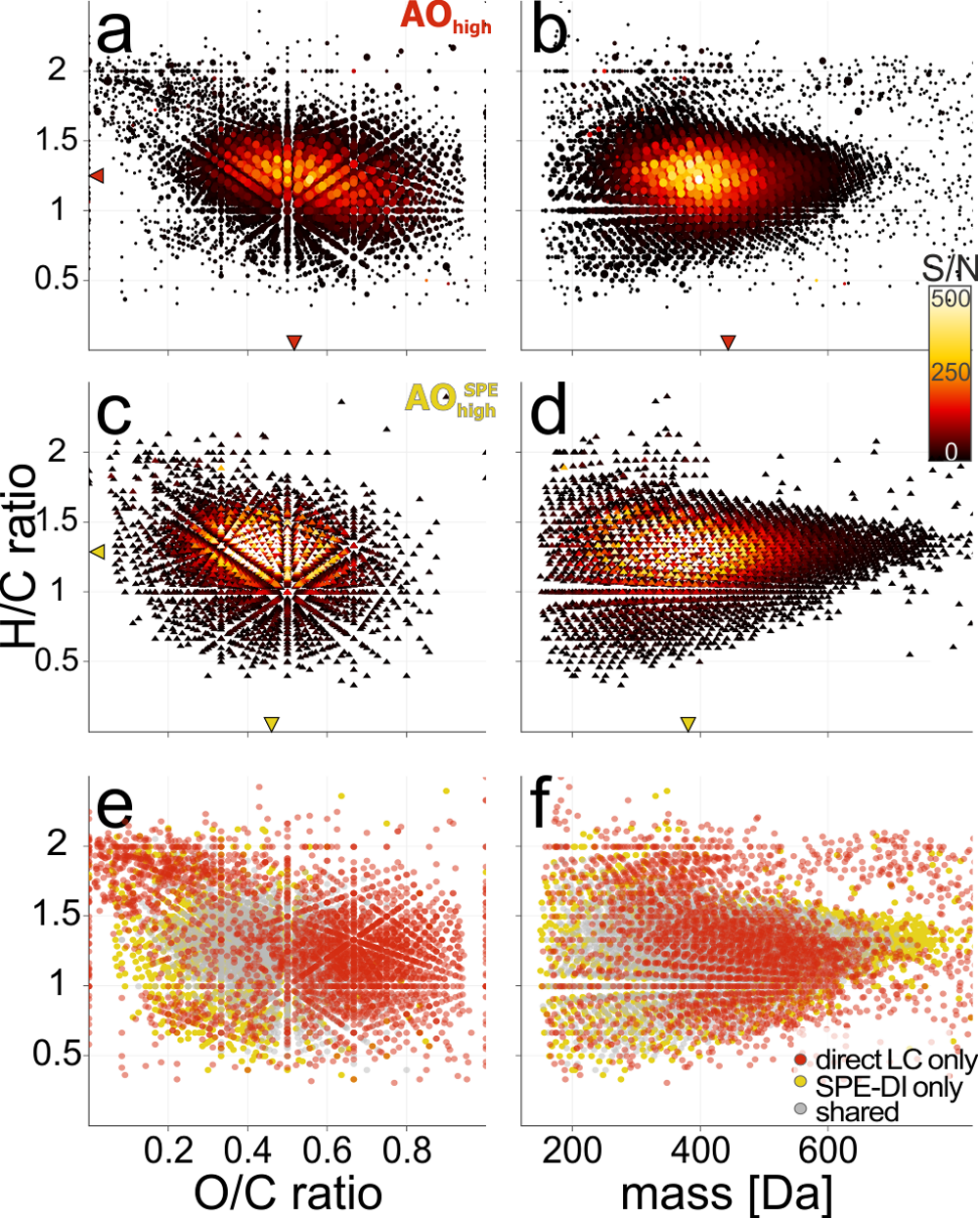
DOM chemodiversity in Arctic Ocean seawater. (a, b) Original sample AO_high_ (circles, *n* = 5,692) measured at 88 μmol DOC L^–1^ with LC-FT-ICR MS and (c, d) the corresponding PPL-extracted sample AO_high_^SPE^ (triangles, *n* = 3,907) measured at 0.8 mmol DOC L^–1^ with DI-FT-ICR MS. Molecular H/C vs O/C (a, c) and H/C vs mass (b, d) for all detected MFs color coded by the signal-to-noise (S/N) ratio. The respective weighted-average values are indicated by markers on the axes. Circle size in (a, b) indicates the number of occurrences (1 ≤ *n* ≤ 9) of each molecular formula across all LC segments (14–24 min). Molecular H/C vs O/C (e) and H/C vs mass (f) for all detected MFs shared (gray, *n* = 2,806) or uniquely detected in original (AO_high_, red, *n* = 2,886), or the PPL extract measured with DI (AO_high_^SPE^, yellow, *n* = 1101).

The state-of-the art method to analyze the molecular composition of seawater DOM is direct infusion (DI) of extracts into FT-ICR MS. We compared DI analysis of AO_high_^SPE^ that was measured with approximately 9-fold enrichment as compared to the original sample (see Methods). While the original water LC analysis of the AO_high_ sample only used 8.8 nmol C and covered 204 LC scans, distributed over 12 min, a larger amount of carbon (27 nmol C) was needed to generate the DI spectrum for which 256 scans were coadded in approximately 7 min. The 9-fold higher concentration, coaddition of a larger number of scans for a single DI-FT-ICR MS spectrum (256 vs 17 for one segment) and corresponding reduction of chemical noise by approximately a factor of 4 contributed to an overall higher sensitivity (dynamic range: 260 with DI after SPE vs 95 with LC from original sample). However, the number of detected MFs was lower for the DI-FT-ICR MS (*n* = 3,907) as compared to the original sample measurement with LC-FT-ICR MS (*n* = 5,692; Figure 6) even without considering multiple detection of the same formula across segments. This confirms that the suppression of low abundance ions (often heteroatom-containing MFs) is reduced due to LC separation, possibly supported by the lower pH of the eluent.^28,44^

The original sample measured with LC displayed a shift toward more oxygenated and less saturated as well as much larger DOM as compared to the DI measurement of its solid-phase extract (Figure 6, Figure SI 7, Table SI 5), as previously observed for SRFA.^28^ In particular, MFs that were uniquely detected in the direct seawater analysis were more polar (i.e., with larger O/C ratio, Figure SI 6) and had a larger N/C and S/C ratio (Table SI 7), confirming the benefits of LC-FT-ICR MS for compounds highly relevant for investigation of the biological processes and carbon cycling.^2,45−47^

At an S/N ratio of 4, we identified 473 MFs in DI-FT-ICR MS measurement of AO_high_^SPE^ that were absent in both LC analyses of the original and extracted sample (Figures SI 30,34). Out of those, 387 MFs had an *S*/*N* < 15 in the DI spectrum and would likely not be detected if measured at the same concentration as the LC analyses. These MFs were characterized by low signal-to-noise, O/C, and H/C ratios in the DI spectrum (Figure SI 34) and hence represented compounds with lower polarity that were preferentially enriched by the PPL extraction.

The comparison between DI-FT-ICR MS of sample AO_high_^SPE^ and LC-FT-ICR MS of AO_high_ indicated more degraded DOM (larger *I*_DEG_) with a lower contribution of terrestrially derived material (smaller *I*_*Terr*_, Table SI 6) in the nonextracted sample. This is notable, since at the same time, a larger relative contribution of terrestrial markers (t-Peaks) to the total intensity was found for the AO_high_ sample as compared to AO_high_^SPE^ (Figures SI 13,14), highlighting uncertainties in the application of biogeochemical indices from DI-FT-ICR MS analyses.

### Biogeochemical Implications

#### Original Water Analysis Revises View on Marine DOM Chemodiversity and Polarity

The current view on marine DOM as assessed with MS is largely based on SPE-extracts known for its consistent underestimation of e.g. the mean nominal oxidation state of carbon (NOSC)^48^ and molecular weight as compared to bulk measurements.^49^ Here we could demonstrate the bias of SPE in marine DOM on a molecular level leading to a predominant detection of less polar DOM, while neglecting a large fraction of polar, heteroatom-rich DOM. Likewise, our results indicate that a substantial fraction of terrestrial-derived DOM was previously overlooked in SPE samples of marine DOM. Such a comparison based on the same detection method was previously not possible, as all molecular-level analyses of marine DOM relied on desalting/extraction and native samples could not be measured.

Our direct analysis of original seawater samples provides a less biased view and allows for a better comparability between samples and with other approaches using original water samples (spectroscopic methods like UV/vis, fluorescence, or FT-infrared spectroscopy). We note that despite the use of nonextracted, original samples, biases from ionization modes and instruments on the observed molecular composition of DOM still exist.^23,50^ For the same reason, the results obtained from our new method cannot be directly compared to DI spectra acquired from extracts. Nevertheless, the biogeochemically most dynamic fraction of DOM (e.g., algal exudates, bacterial exometabolites, terrestrial DOM)^5,51,52^ can now be better studied due to the increased sensitivity from the polarity separation and reduced suppression of heteroatom-containing DOM.

#### New Perspectives for DOM Research

The small volume requirements of the new method support studies where only a small sample volume is available (e.g., in sediment pore water) and also allow higher spatial (e.g., in soils) or temporal sample resolution (e.g., in culture experiments). The sensitivity of the method is unprecedented since the lowest absolute amount of carbon injected was only 2 nmol C (20 μmol DOC L^–1^). Given about 5,700 molecular formulas and 16,200 detected molecular features in a marine DOM sample and an average number of carbon atoms of 19, the mean absolute detectable amount for a molecular feature was around two femtomole. The linear response of the peak magnitudes now allows DOM characterization beyond the compositional analyses that uses normalized peak magnitudes. Instead, we can now use the peak magnitude directly or, alternatively, DOC concentration as the normalization factor for semiquantitative evaluations of MF abundances. In combination with the isomeric separation based on polarity, this method now allows revisiting controversial concepts regarding the long-term stability of marine organic matter and the fate of terrestrial organic matter in the ocean.^53,54^

Compared to DI analyses, the use of LC leads to a significant increase in the amount of data (up to 20 Gigabyte per sample with full profile mode and retaining the free induction decay for about 20–25 min data recoding time). This requires revised concepts for automated data processing^55^ and quality control as well as statistical analysis. These additional efforts are justified given the less biased analysis and greater information gain, especially on the chemistry of DOM. Further improvements in sensitivity may also allow nontargeted environmental metabolomics studies performed with original seawater. Ultimately, we expect that novel biomarkers can be developed, making use of the high analytical sensitivity obtained from LC-FT-ICR MS to study environmental processes.

## Data Availability

Processed and quality checked data for all samples and segments are available from the UFZ Data Investigation Portal: https://doi.org/10.48758/ufz.14331. Raw MS files can be shared upon request.

## Supporting Information Available

Additional information regarding DOC determination, instrument quality control, and method assessment, samples (Tables SI 1,2, Figure SI 1), model compounds (Tables SI 3,4, Figure SI 16), molecular descriptors and biogeochemical indices (Tables SI 5–7), LC setup (Figure SI 2), data processing (Figure SI 3), LC chromatograms (Figures SI 4–6, 12–15), chemodiversity (Figures SI 7–11), matrix effects (Figures SI 17–21), detector response (Figures SI 22–24), repeatability (Figures SI 25,26), pH effect (Figures SI 27,28), comparison with SPE extracts (Figures SI 29–34) (PDF)
Script for DataAnalysis (Bruker Daltonics, version 6.0) for LC-MS segmentation and spectra averaging (TXT)
List of surfactants (XLSX)
