# Supplementary material for: Direct Analysis of Marine Dissolved Organic Matter Using LC-FT-ICR MS
Source: Environ Sci Technol. 2024 Mar 1;58(10):4637–47. doi: 10.1021/acs.est.3c07219 (PMC10938638; doi:10.1021/acs.est.3c07219)
Supplement: Supplementary file 1 — es3c07219_si_001.pdf [file es3c07219_si_001.pdf]

Supporting Information for:

Direct analysis of marine dissolved organic matter using LC-FT-ICR MS

Oliver J. Lechtenfeld[1,2,*], Jan Kaesler[1], Elaine K. Jennings[1], Boris P. Koch[3,4*]

[1] Helmholtz Centre for Environmental Research – UFZ, Department of Environmental Analytical Chemistry, Research Group BioGeoOmics, Permoserstraße 15, 04318 Leipzig, Germany

[2] ProVIS–Centre for Chemical Microscopy, Helmholtz Centre for Environmental Research − UFZ, Permoserstraße 15, 04318 Leipzig, Germany

[3] Alfred-Wegener-Institut Helmholtz-Zentrum für Polar- und Meeresforschung, Am Handelshafen 12, 27570 Bremerhaven, Germany

[4] University of Applied Sciences, An der Karlstadt 8, 27568 Bremerhaven, Germany

* Corresponding authors (oliver.lechtenfeld@ufz.de, boris.koch@awi.de)

No. of pages: 39

No. of tables: 7

No. of figures: 34

Table of Contents

SI Text 3

DOC determination 3

Instrument Quality Control 3

Method Assessment 4

SI Tables 7

Samples 7

Model Compounds 9

Molecular descriptors and biogeochemical indices 10

SI Figures 12

Sample overview 12

LC-FT-ICR MS Setup and Gradient 13

Data processing 14

Total and Extracted Ion Chromatograms 15

DOM Chemodiversity 18

Marine and terrestrial marker in LC-FT-ICR MS 22

Salt separation on the LC column 25

Model Compounds Intermediate Precision 26

Matrix Effects 27

Detector Response and Linearity 31

Repeatability 33

Sample pH Effect 34

Original Water vs. SPE 35

Original Water vs. DI 38

Additional references 39

SI Text

DOC determination

DOC concentrations for original Arctic Ocean samples (AO_{high}, AO_{low}) were determined by high temperature catalytic oxidation (HTCO) and non-dispersive infrared spectroscopy detection (TOC-VCPN, Shimadzu). Samples were poured into well-rinsed vials and acidified with 0.1 M HCl Suprapur (Merck) in the autosampler. Oxygen was then sparged into the samples for 5 minutes to remove inorganic carbon before 50 µL sample volume was injected directly on the catalyst at 680°C. The CO_2 generated was detected using an infrared detector, with a limit of determination of 7 µmol DOC L^{-1} and a repeatability of ±5%. DOC concentration of the PPW sample and the SPE extract of samples AO_{high} (AO_{high}^{SPE}) was determined with the HTCO method (DIMATOC 2100, Dimatec Analysentechnik, Essen, Germany) in accordance with EN 1484 (TOC) at UFZ Leipzig.

Instrument Quality Control

For instrument quality control, Suwannee River Fulvic Acid standard (SRFA, 2S101H, International Humic Substances Society) were reconstituted in ultra-pure water to yield a carbon concentration of 0.8 mmol DOC L^{-1} (10 mg L^{-1}). The complex DOM standard was used to monitor mass spectral performance (sensitivity, peak shape). To monitor chromatographic robustness which may also be affected by the high salt content, standard compounds ("model compounds", MC; 2-(4-(2,2-dicarboxy-ethyl)-2,5-dimethoxy-benzyl)malonic acid, isoferulic acid 3-O-beta-D-glucuronide, vanillic acid, leu-enkephalin, fraxin, all from Sigma-Aldrich) were added to SRFA so that extracted ion intensities match DOM m/z intensities (cf. Han et al. (2021); Patriarca et al. (2017)). The MC retention times approximately covered the elution range of DOM (Table SI 3).

After each set of ten samples, a pure water blank (to examine carry-over) and SRFA standard spiked with MCs was injected. For the spiked SRFA, MC signals were extracted from the LC-FT-ICR MS data (using DataAnalysis, version 5.0, Bruker) and their retention time and peak area analyzed to assess the

impact of the high salt loading on the performance of the LC column within a (Table SI 3). The stability of the chromatographic system was evaluated during a long sequence with > 130 salt-containing samples using the same LC column (Table SI 4).

Method Assessment

Linear detector response and sensitivity. The linearity of the ion detection in the ICR cell was determined with PPW injected at different concentrations (20, 40, 80, 160 µmol DOC L^{-1}) covering the typical seawater DOC concentration range (PPW^{S0} samples). The number of assigned MFs and the total sum of all peak magnitudes that were assigned with a formula (total assigned intensity) were used to assess the method sensitivity. A linear regression based on peak magnitudes was calculated for all MFs detected at all concentration levels. Pearsons r^2 and corresponding *p*-values were used to evaluate the ICR detector linearity. The effect of increasing ion abundances on DOM composition was checked with the aggregated molecular descriptors.

Robustness. The effect of salt on the DOM mass spectra was assessed in two ways: Firstly, 35 g L^{-1} NaCl (PPW^{S35} samples) was added to the PPW sample prepared at different concentrations (40, 80, 160 µmol DOC L^{-1}). This set of samples is comparable to the samples used to assess the linear range, but additionally accounts for sensitivity effects due to residual salt. It was used to evaluate the loss of polar DOM from the analysis due to superimposed salt elution (this fraction is directed to waste for the seawater sample). The data were further evaluated based on the number of molecular formulas, total assigned intensity, and aggregated molecular descriptors for each segment. In addition, the difference in the RAW peak intensity (raw mass peak magnitudes), calculated as $\delta RAW = (RAW^{S35} - RAW^{S0})/RAW^{S0}$, and the slopes of the linear regression of RAW peak intensities with DOC concentration were used to assess suppression from co-eluting salt.

Secondly, 17 g L^{-1} NaCl was added to a diluted (80 µmol DOC L^{-1}) PPW sample (PPW^{S17} samples) and evaluated together with the PPW^{S0} and PPW^{S35} samples at the same DOC concentration to mimic

a freshwater-estuarine-seawater-sea ice formation gradient. This set of samples was used to evaluate matrix effects and impact of residual salts.

Repeatability and intermediate precision: Repeatability was assessed with a seawater sample (AO_{low}) that was measured in triplicate within a sample sequence. The number and sum of intensity of molecular formulas that were shared across the triplicates in a retention time segment were evaluated. Further, the reproducibility of RAW peak intensities of shared MFs across all replicates was calculated as coefficient of variance (CV). In addition, the intermediate precision of the method was assessed as CV of the peak area of five MCs spiked into SRFA and repeatedly (n = 3) measured during the sequence as well as in a sequence comprising > 130 seawater samples analysed over the course of 5 days (n = 11).

Comparison with PPL extracts. Finally, a comparison with state-of-the art methods was performed by comparing the PPL extracted sample (AO_{high}^{SPE}) measured with LC-FT-ICR MS (diluted to original concentration) and direct infusion (DI-) FT-ICR MS (at 10 mg DOC L^{-1} equivalent to 0.83 mmol DOC L^{-1}). For the LC-FT-ICR MS comparison, the number of (shared) molecular formulas, the total assigned intensity, and the aggregated molecular descriptors for each segment were evaluated. In addition, the relative difference in the RAW peak intensity, was calculated as $\delta RAW = (RAW[AO_{high}] - RAW[AO_{high}^{SPE}])/RAW[AO_{high}]$ to evaluate the effect of extraction on the observable molecular composition.

In addition, we compared the original and SPE-extracted sample based on the total assigned intensity and number of molecular formulas of all segments. Since the PPL extracted sample did not contain salt, the early eluting DOM fraction (6.5 min - 13.5 min), as recovered by PPL, was included for this comparison. Further, for a comparison of the LC-FT-ICR MS data (both direct and extracted) with DI-FT-ICR MS data, an intensity averaged pseudo-DI measurement was prepared using all MFs present

in any LC segment. MFs shared between the three samples were evaluated based on their number and molecular descriptors.

SI Tables

Samples

Table SI 1: Sample origin and basic chemical parameters. AO = Arctic Ocean, PPW = peat pore water.

Sample name<br>Sample ID	Source	Sampling Date	DOC [µmol L^{-1}]	Salinity	Lat [°]	Long [°]	Depth [m]
AO_{low}<br>PS122_DOC_487	Arctic Ocean	2020-04-30	55[†]	34.3	83.9385	17.4027	2.6
AO_{high}[§]<br>PS122_DOC_390	Arctic Ocean	2020-03-27	88[†]	33.7	85.8241	13.3624	2.1
PPW	Peat pore water	2015	21250[‡,#]	0[#]	52.5945	8.6721	0.1

[†] Dissolved organic carbon (DOC) concentrations of the AO samples were determined by high-temperature catalytic oxidation (HTCO), nondispersive infrared spectroscopy (TOC-VCPN, Shimadzu) at AWI Bremerhaven.

[§] The AO_{high} sample was also extracted with SPE, cf. Table SI 2.

[‡] DOC concentration of the PPW sample was determined with the HTCO method (DIMATOC 2100, Dimatec Analysentechnik, Essen, Germany) at UFZ Leipzig.

[#] The DOC concentration and salinity of the PPW sample was adjusted with ultra-pure water and NaCl, cf. Table SI 2 and Figure SI 1.

Table SI 2: Samples used in this study. Basic chemical parameters (DOC concentration and salinity), main text chapter and purpose during method validation.

Sample name	DOC [µmol L^{-1}]	Salinity	Described in chapter… *(Data in main text figure…)*		Used for testing…
AO_{low}	55	34.3	Chemodiversity and polarity of marine DOM *(Figure 1, Figure 2)*	Repeatability and intermediate precision	Repeatability (triplicate injections)
AO_{high}	88	33.7		Effect of PPL extraction on the observable DOM chemodiversity *(Figure 5)* Comparison of conventional direct infusion and original water LC analysis *(Figure 6)*	Comparison with PPL extracts
AO_{high}^{SPE}	88[‡]/800[‡,§]	n.d.			
AO_{low}^{pH3}	55	34.3	Recommendations for sample and data handling		Robustness
PPW_{20}^{S0}	20	n.d.	Linear detector response *(Figure 4)*		Linear detector response and sensitivity
PPW_{40}^{S0}	40	n.d.			
PPW_{80}^{S0}	80	n.d.		Enabling direct seawater DOM analysis with LC-FT-ICR MS *(Figure 3)*	
PPW_{160}^{S0}	160	n.d.			
PPW_{40}^{S35}	40	35			i) Linear detector response and sensitivity ii) Robustness
PPW_{80}^{S35}	80	35		Enabling direct seawater DOM analysis with LC-FT-ICR MS *(Figure 3)*	
PPW_{160}^{S35}	160	35			
PPW_{80}^{S17}	80	17			Robustness
PPW_{160}^{S70}	160	70			
SFRA with spiked model compounds	800	n.d.		Repeatability and intermediate precision	Intermediate precision (11 injections)

n.d. = not determined.

[§] for DI-FT-ICR MS.

[‡] DOC concentration of the AO_{high} MeOH extract after SPE was 9.2 mmol DOC L^{-1}, determined with the HTCO method (DIMATOC 2100, Dimatec Analysentechnik, Essen, Germany) at UFZ Leipzig.

Model Compounds

Table SI 3: Model compound repeatability. LC retention time [min], peak full width at half maximum [min] and MS peak area and S/N values calculated for three repeat injections of SRFA (10 mg L^{-1}) spiked with model compounds. Values are provided as mean ± standard deviation.

Index	Model compound name	Concentration [ng mL^{-1}]	Retention time [min]	FWHM [min]	Area	CV area [%]	S/N
1	2-(4-(2,2-Dicarboxy-ethyl)-2,5-dimethoxy-benzyl)malonic acid	40	17.60 ± 0.01	0.25 ± 0.00	846 462 933 ± 52 272 941	6	67 ± 10
2	Isoferulic acid 3-*O*-β-D-glucuronide	40	18.12 ± 0.02	0.30 ± 0.01	801 687 531 ± 51 321 222	6	47 ± 6
3	Vanillic acid	500	18.64 ± 0.01	0.21 ± 0.02	162 900 627 ± 42 311 778	26[§]	51 ± 13
4	Fraxin	80	19.35 ± 0.04	0.26 ± 0.02	304 340 299 ± 15 546 640	5	32 ± 9
5	Leu-enkephalin	50	19.50 ± 0.03	0.26 ± 0.02	133 937 904 ± 7 050 820	5	35 ± 1

[§] This compound was excluded from the repeatability assessment due to an outlier injection.

Table SI 4: Model compound long term repeatability/intermediate precision. LC retention time [min] and MS peak area calculated for 11 repeat injections of SRFA (10 mg L^{-1}) spiked with model compounds in a sequence spanning > 130 seawater samples. Values are provided as mean ± standard deviation. Note that different MC spike levels were used as compared to Table SI 3, resulting in different mean peak areas.

Index	Model compound name	Retention time [min]	Area	CV area [%]
1	2-(4-(2,2-Dicarboxy-ethyl)-2,5-dimethoxy-benzyl)malonic acid	17.40 ± 0.02	1 797 243 287 ± 162 107 587	9
2	Isoferulic acid 3-*O*-β-D-glucuronide	17.93 ± 0.03	1 819 879 645 ± 157 334 938	9
3	Vanillic acid	18.48 ± 0.02	126 382 863 ± 21 887 970	17
5	Fraxin	19.25 ± 0.02	854 124 399 ± 105 281 116	12
4	Leu-enkephalin	19.13 ± 0.05	419 590 182 ± 71 277 478	17

Molecular descriptors and biogeochemical indices

Table SI 5. Average molecular descriptors. All descriptors from LC-FT-ICR MS measurements were calculated from MFs intensities from intensity-averaged pseudo-DI spectra considering segments from 14 - 22 min. "wa" refers to intensity-weighted average descriptors.

Sample	*m/z*	O/C_{wa}	H/C_{wa}	N/C_{wa} (x1000)	S/C_{wa} (x1000)	MF	CHO (%)	CHNO (%)	CHOS (%)	CHNOS (%)
AO_{high}	449	0.52	1.26	32	7.4	5692	2362 (41.5)	2064 (36.3)	621 (10.9)	589 (10.3)
AO_{high}^{SPE}	460	0.48	1.24	23	4.2	6607	3188 (48.3)	2376 (36.0)	528 (8.0)	475 (7.2)
AO_{high}^{SPE}-DI	420	0.44	1.28	12	1.2	3907	2270 (58.1)	1344 (34.4)	222 (5.7)	68 (1.7)
AO_{low}	449	0.52	1.28	32	7.4	5802	2468 (42.5)	2048 (35.3)	624 (10.8)	610 (10.5)

Table SI 6. Biogeochemical indices. I_{DEG} (Flerus et al. (2012)), IOS (Lechtenfeld et al. (2014)), I_{Terr} and t-Peaks (Medeiros et al. (2016)). All indices from LC-FT-ICR MS measurements were calculated from MFs intensities from intensity-averaged pseudo-DI spectra considering segments from 14 - 22 min.

Sample	$I_{DEG}^{\dagger}$	# I_{DEG}-MF (NEG – POS)	# IOS-MF	$I_{Terr}^{\S}$	# I_{Terr}-MF (*Terr* – *Mar*)	# t-Peaks
AO_{high}	0.60	5/5 – 5/5	360/361	0.08	39/40 – 39/40	147/184
AO_{high}^{SPE}	0.57	5/5 – 5/5	361/361	0.12	39/40 – 39/40	172/184
AO_{high}^{SPE}-DI	0.45	5/5 – 5/5	357/361	$0.16^{\#}$	39/40 – 39/40	179/184
AO_{low}	0.60	5/5 – 5/5	360/361	0.08	39/40 – 39/40	149/184

† ∑NEG/∑(NEG+POS), according to Flerus et al., 2012.[3]

§ ∑*Terr*/∑(*Terr*+*Mar*), according to Medeiros et al., 2016.[5]

\# Molecular formula $C_{16}H_{16}O_5$ (*Terr*) and $C_{19}H_{24}O_9$ (*Mar*) were removed from the final data set for the DI measurement and thus not considered for all other measurements.

Table SI 7. Molecular descriptors of shared and unique molecular formulas (MF). All descriptors from LC-FT-ICR MS measurements were calculated from MF intensities from intensity-averaged pseudo-DI spectra considering segments from 14 - 22 min. Here, the two LC-FT-ICR MS (AO_{high} and AO_{high}^{SPE}) and DI-FT-ICR MS measurement of AO_{high}^{SPE} were compared. The breakdown of shared and unique MF is shown in Figure SI 30. "wa" refers to intensity-weighted average descriptors.

Group	*m/z*	O/C_{wa}	H/C_{wa}	N/C_{wa} (x1000)	S/C_{wa} (x1000)	MF	CHO	CHNO	CHOS	CHNOS
shared across all	408	0.45	1.28	11.5	0.9	2753	1568	1019	12	154
shared SPE+DI and direct LC	284	0.42	1.49	22.9	1.8	53	28	19	3	3
shared SPE (DI + LC)	465	0.35	1.23	15.4	4.5	628	421	158	7	42
shared LC (SPE + direct)	435	0.59	1.26	44.0	9.9	1198	453	515	97	124
unique direct LC	530	0.56	1.33	47.9	27.9	1688	313	511	477	340
unique SPE+LC	545	0.47	1.22	35.2	13.8	2028	746	684	359	208
unique SPE+DI	447	0.34	1.37	20.3	5.3	473	253	148	46	23

SI Figures

Sample overview

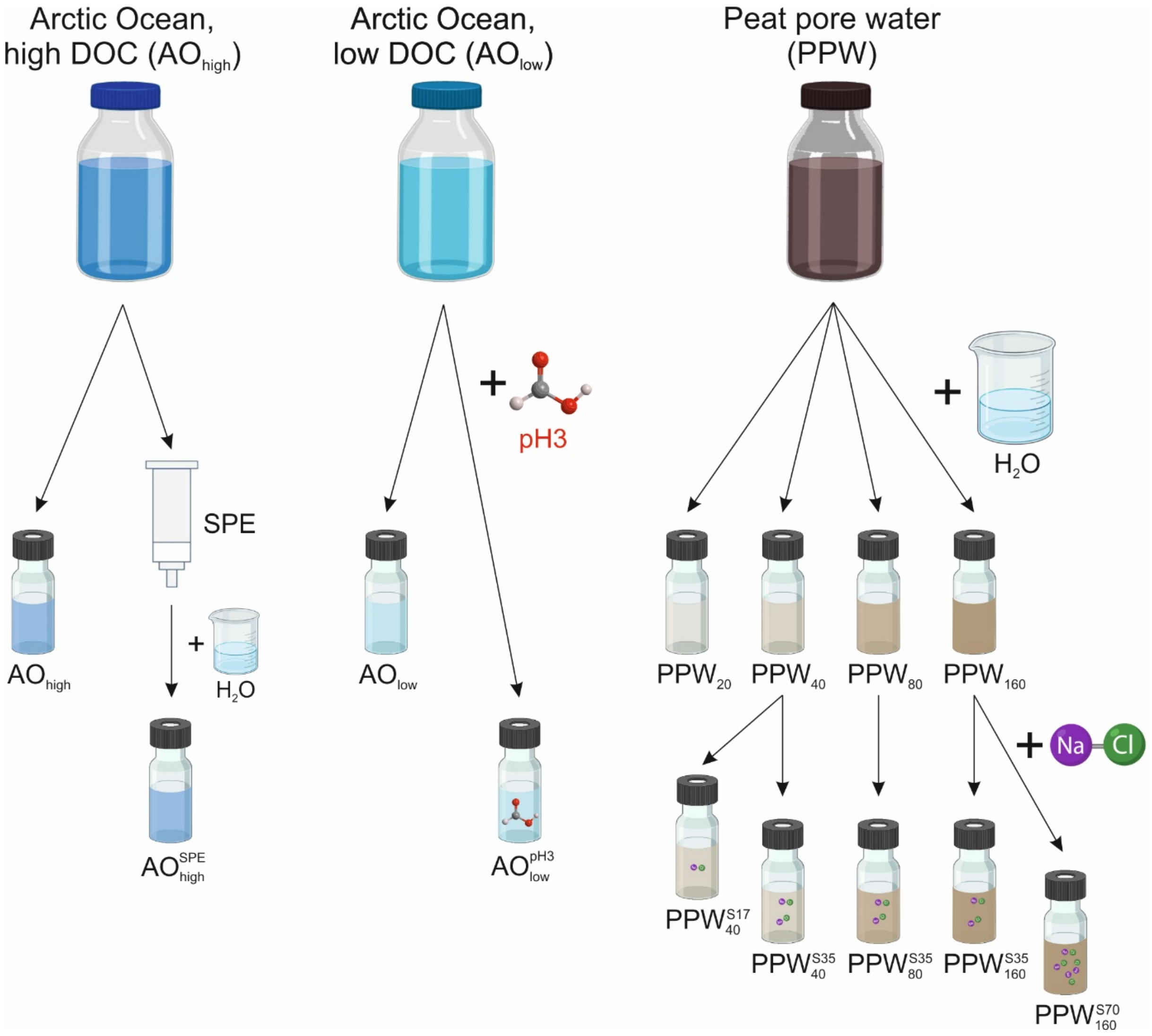

Figure SI 1: Overview of samples used in this study. Two original seawater samples (AO_{high} and AO_{low}) were used for initial LC-FT-ICR MS method testing. For the comparison with state-of-the art SPE-based methods, the AO_{high} sample was extracted using PPL (AO_{high}^{SPE}) and measured with LC-FT-ICR MS and DI-FT-ICR MS. To test the effect of sample pH, formic acid was added to sample AO_{low} (AO_{low}^{pH3}) to match the pH of the eluent. A peat pore water (PPW) sample was diluted with ultrapure water to different target DOC concentrations (PPW_{20-160}) and NaCl was added to an aliquot to simulate the salinity of seawater (PPW_{40-160}^{S35}), estuarine water (PPW_{80}^{S17}), and sea ice brine (PPW_{160}^{S70}). Parts of the figure were created using biorender.com.

LC-FT-ICR MS Setup and Gradient

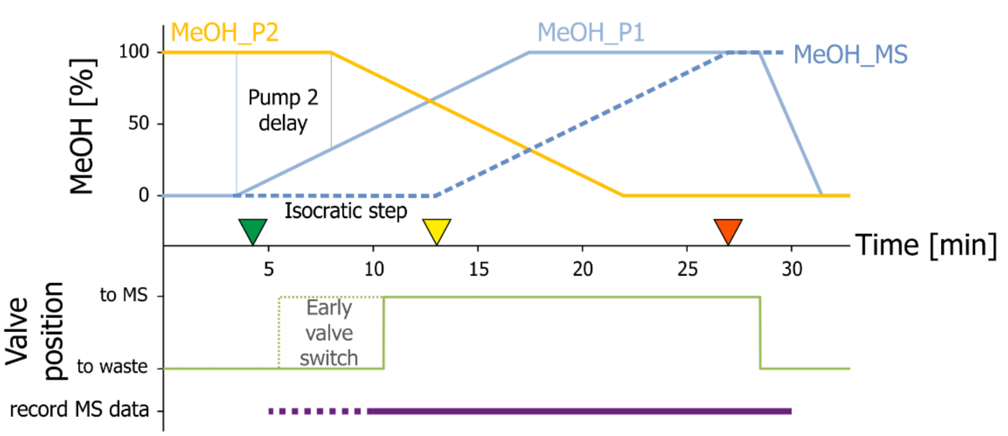

Figure SI 2: Gradient program of the liquid chromatography (LC) system. The methanol gradient for the main pump (MeOH_P1, light blue) and the auxiliary pump (MeOH_P2, yellow) is indicated as solid line. The difference between the start time of the gradient for both pumps is the “Pump 2 delay” and mainly caused by the additional LC-column void volume in the flow path before the T-junction. Due to the total void volume and flow rate in the system (4.8 min, indicated by the green marker), the MeOH gradient from the main pump is first detected at the MS at approx. 13 min (yellow marker), resulting in a shift of the gradient at the ICR detector (dashed blue line). The time between the void volume and the start of the gradient is the “isocratic step”. 100% methanol reached the MS at 27 min (red marker). For seawater injections, the 6-port-2-way valve between LC and MS is switched to waste until 10.5 min (green line) and the MS is recorded between 10 and 30 min (purple line). For non-seawater tests, valve switch and MS recording start time was shifted to 5.5 and 5 min, respectively.

Data processing

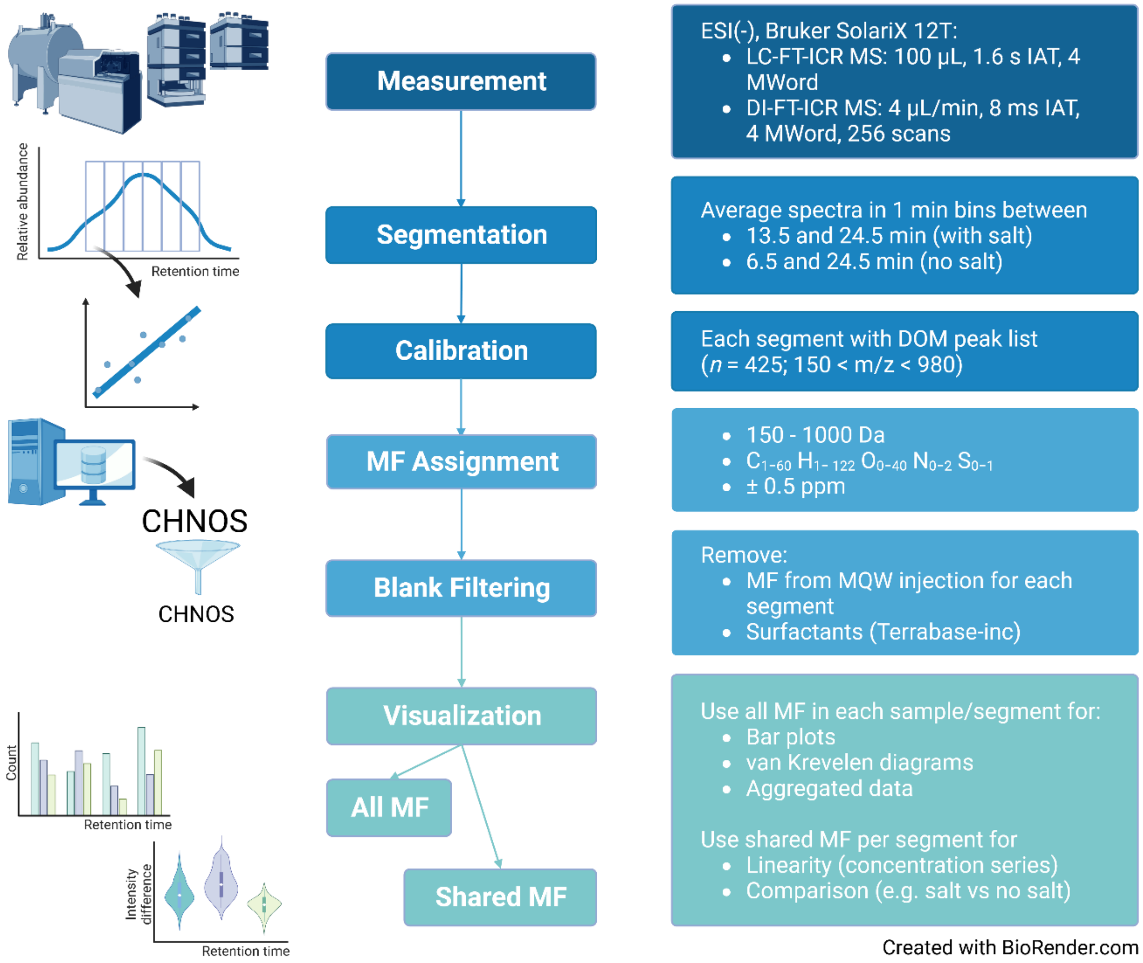

Figure SI 3: Overview of the complete data processing as described in the main text.

Total and Extracted Ion Chromatograms

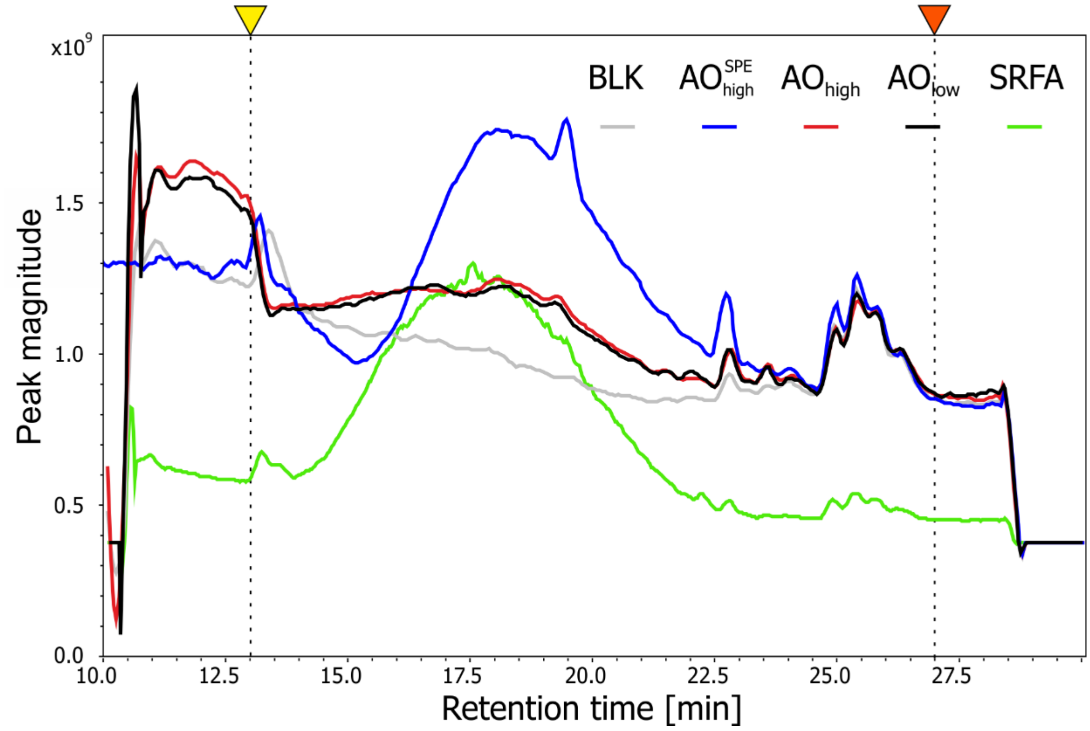

Figure SI 4: Total ion chromatograms (TIC, solid lines) for all samples used in this study. The yellow and red marker and dashed line indicate the first (13 min) and 100% methanol (27 min) from the gradient. Note that due to the low concentration of DOM and high accumulation time used, the majority of the TIC is noise (high baseline in mass spectrum, as evident from the blank injection). Note that SRFA (green) was measured with 0.5 s ion accumulation time, resulting in a lower baseline intensity.

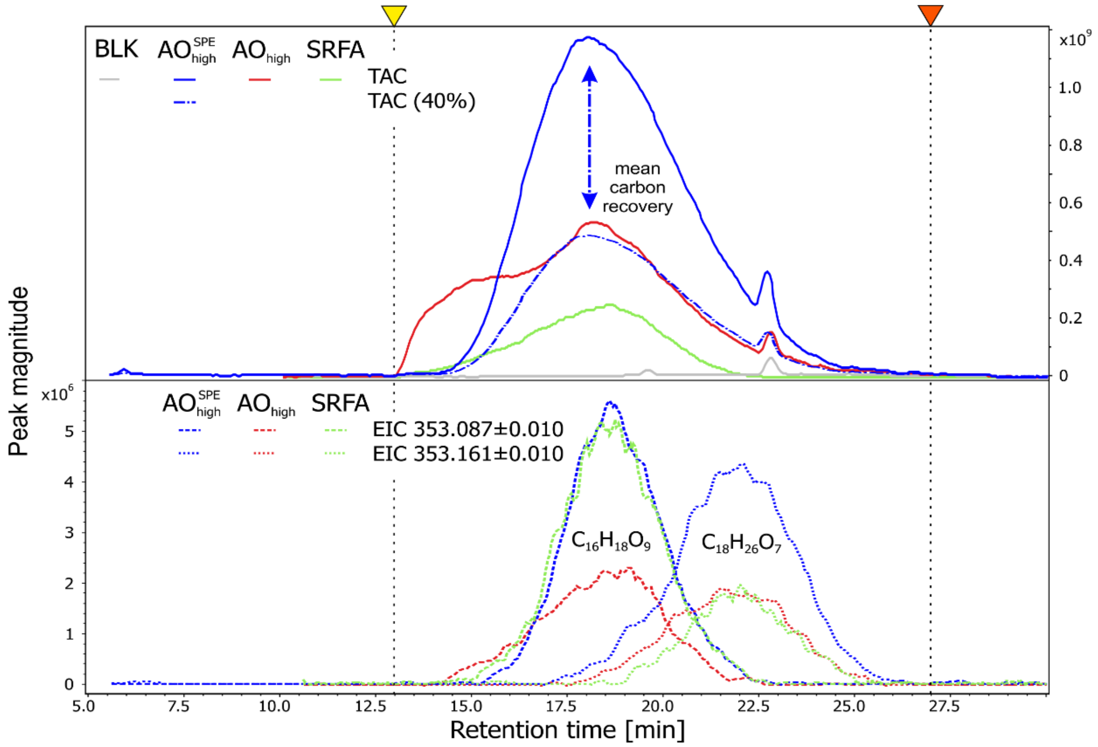

Figure SI 5: Total assigned ion chromatograms (TAC, upper panel, solid lines, right y-axis) and extracted ion chromatograms (EIC, lower panel, dashed lines, left y-axis) for the Arctic Ocean samples AO_{high} (red), its SPE extract AO_{high}^{SPE} (blue) and SRFA (green). For the SPE extract AO_{high}^{SPE}, an adjusted TAC is displayed (upper panel, blue dash-dot line) that considers the loss in observable peak magnitude due to incomplete bulk carbon SPE recovery (~40%, cf. Figure 5 in the main text). Two m/z values having the same nominal mass (lower panel) represent molecular formulas with high O/C (m/z 353.087: $C_{16}H_{18}O_9$) and low O/C ratio (m/z 353.161: $C_{18}H_{26}O_7$). The yellow and red marker and dashed line indicate the first (13 min) and 100% methanol (27 min) of the gradient. Note that SRFA (green) was measured with 0.5 s ion accumulation time, resulting in a lower summed peak magnitude for TAC.

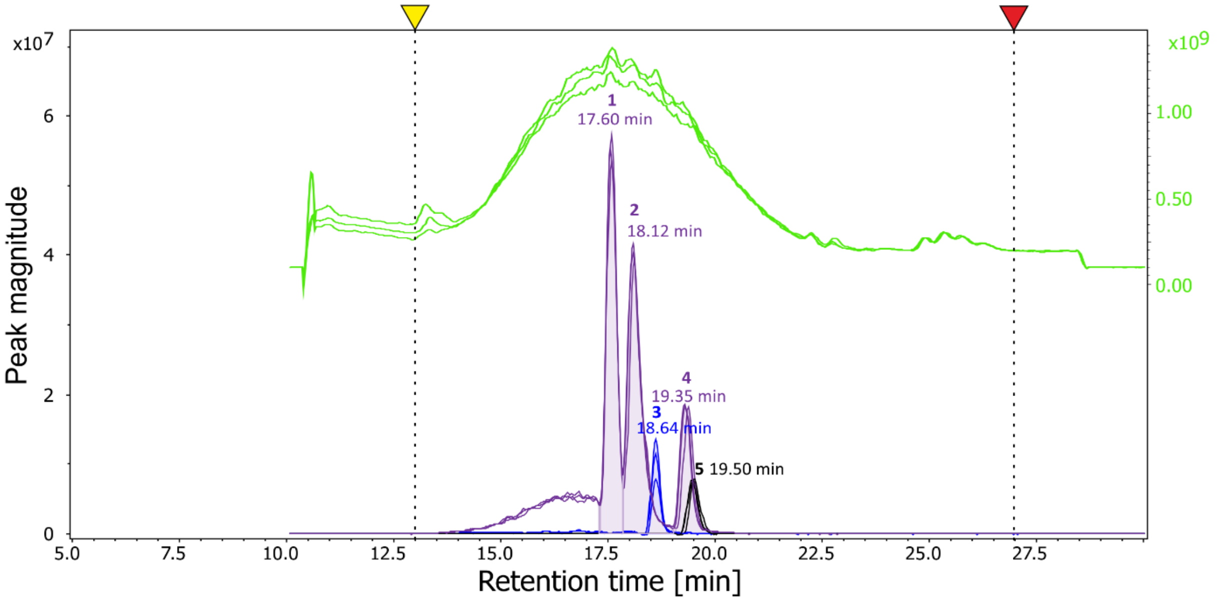

Figure SI 6: Model compounds in SRFA. Extracted ion chromatograms (m/z 167.035±0.005 (blue), m/z 369.083±0.005 (purple), m/z 554.262±0.005 (black), left y-axis) for model compounds and the total ion chromatogram (TIC, green, right y-axis) for three injections of SRFA (10 mg L^{-1}) spiked with model compounds. The bold numbers refer to the model compound in Table SI 3. Note that m/z 369 is also present in SFRA, indicated by the background "hump". Chromatograms were smoothed with the Savitzky-Golay method (9 points, 1 cycle). The yellow and red marker and dashed line indicate the time when the first and 100% methanol reached the MS.

DOM Chemodiversity

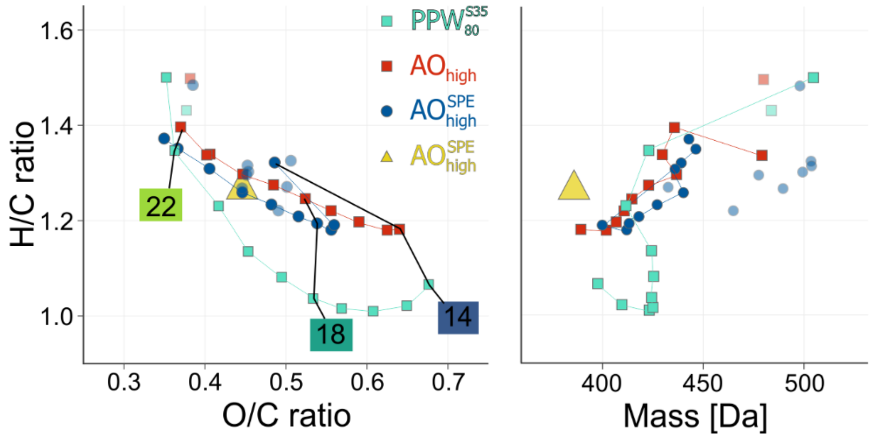

Figure SI 7: Mean chemical characteristics for samples AO_{high}, AO_{high}^{SPE}, and PPW. Intensity-weighted aggregated molecular H/C and O/C ratios (right) and mass (left) for all segments of three samples measured at similar DOC concentrations (AO_{high}: 88 µmol DOC L^{-1}, red; AO_{high}^{SPE}: 88 µmol DOC L^{-1}, blue; PPW with 35 g L^{-1} NaCl: 80 µmol DOC L^{-1}, turquoise) and AO_{high}^{SPE} (yellow) measured with DI at 0.8 mmol DOC L^{-1}. Note that the segments between 7 and 14 min (AO_{high}^{SPE}) and at 24 min only contained very few MFs (transparent squares). The lines are for reference to the same segments and same sample only. Detailed molecular composition for selected segments of samples AO_{high} and AO_{high}^{SPE} are displayed in Figure SI 10, and Figure SI 11, respectively.

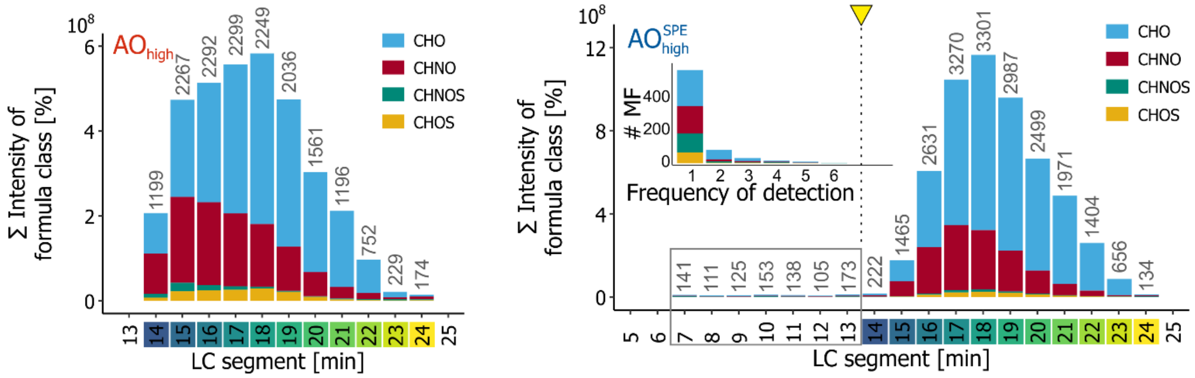

Figure SI 8: Segment-wise intensity and formula class distribution in original seawater and extract. Summed intensity of assigned molecular formulas (MFs) based on formula classes (colors in legend) for the AO_{high} (left) and AO_{high}^{SPE} (right) sample with all 11 (14 - 24 min) and 18 (7 – 24 min) segments, respectively. For the seven early segments in sample AO_{high}^{SPE} (7 – 13 min) the frequency of detection is shown as insert (cf. Figure SI 29). The yellow marker and dashed line indicate the retention time at which the first methanol reaches the MS (13 min).

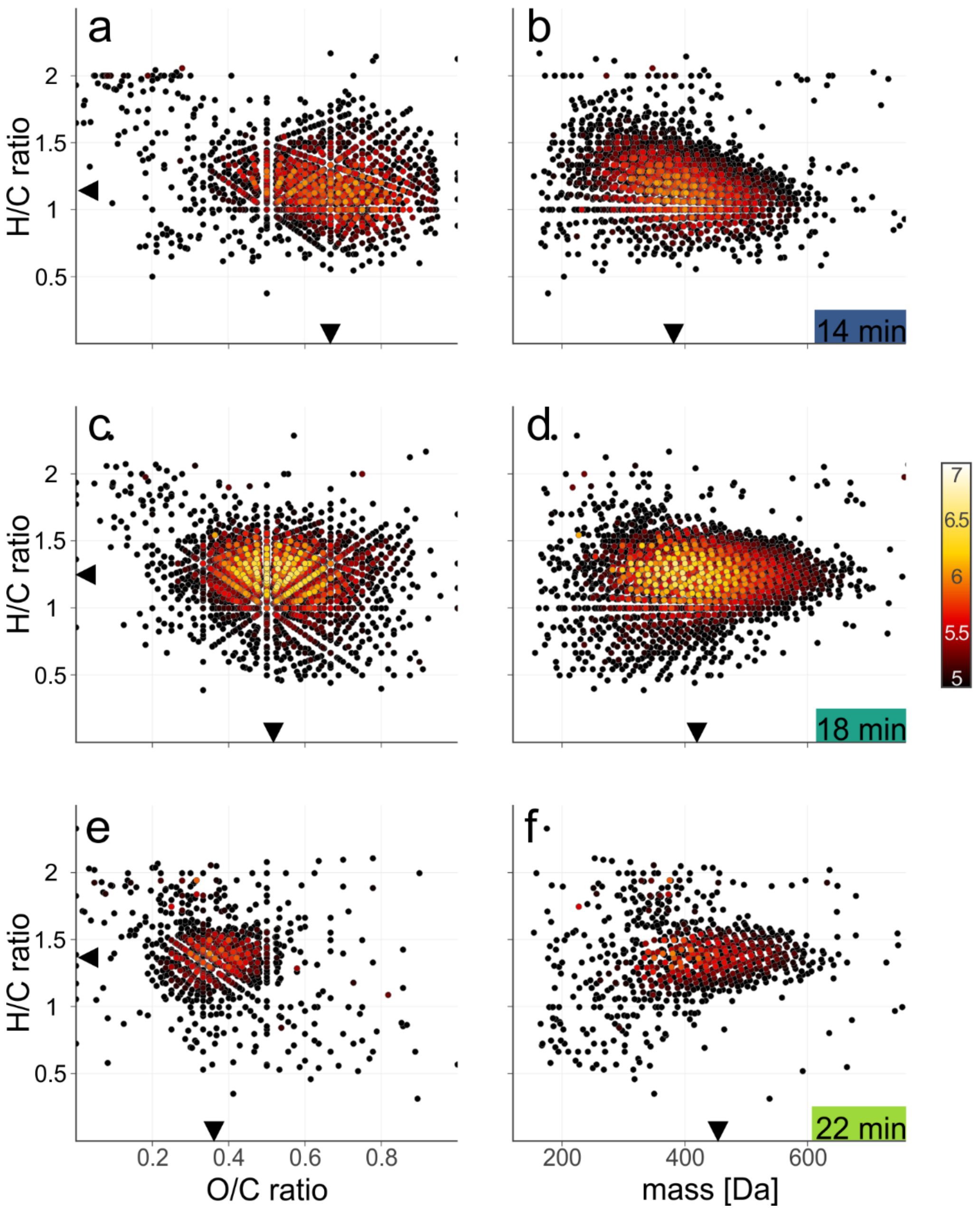

Figure SI 9: DOM chemodiversity in sample AO_{low}. Molecular H/C vs. O/C (a, c, e) and H/C vs. mass (b, d, f) for all detected molecular formulas in selected segments at 14 min (a, b), 18 min (c, d) and 22 min (e, f), color coded by absolute intensity (log10). The respective weighted aggregated values are indicated by black markers on the axes.

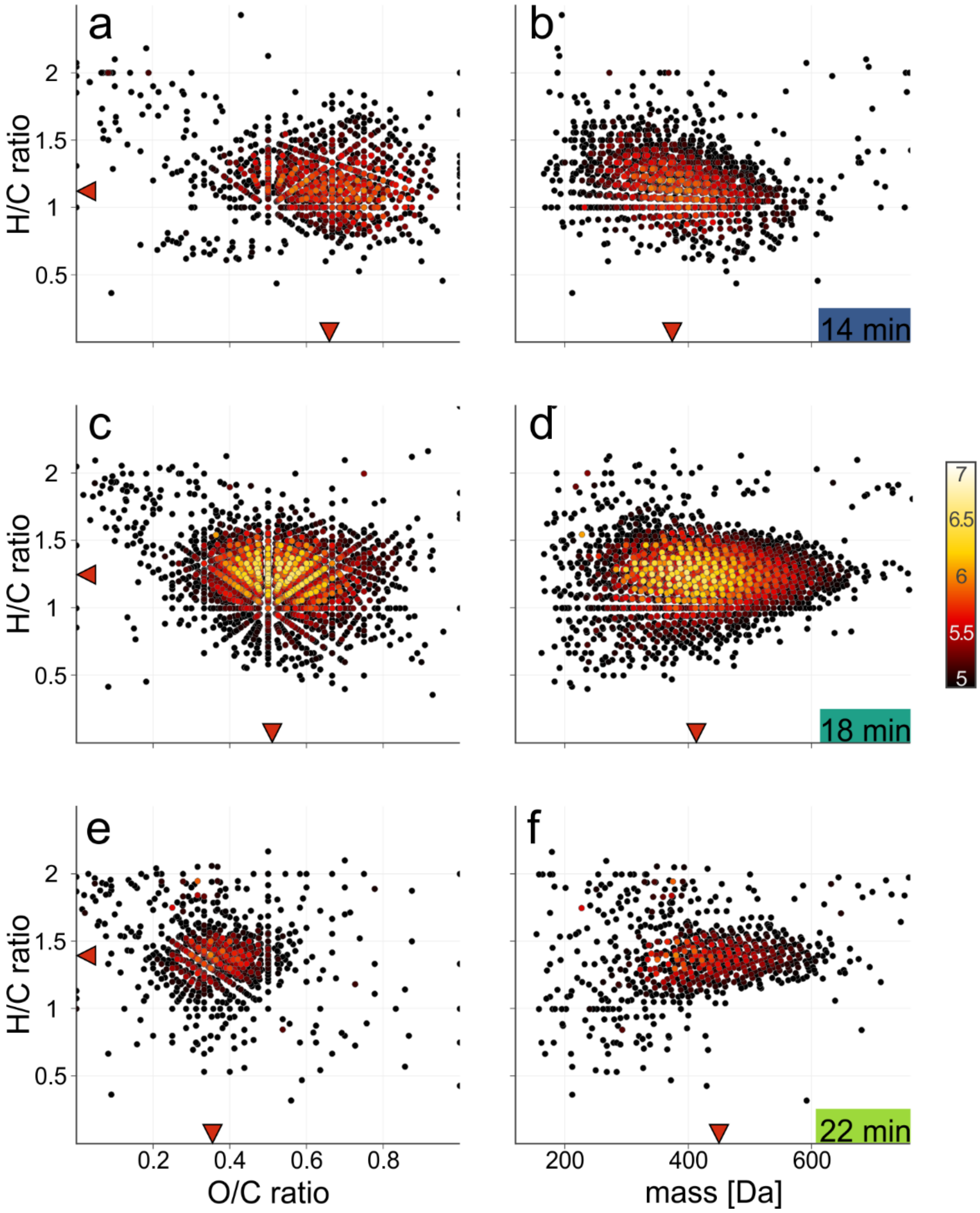

Figure SI 10: DOM chemodiversity in sample AO$_{high}$. Molecular H/C vs. O/C (a, c, e) and H/C vs. mass (b, d, f) for all detected MFs in selected segments at 14 min (a, b), 18 min (c, d) and 22 min (e, f), color coded by absolute intensity (log$_{10}$). The respective weighted-average values are indicated by red markers on the axes.

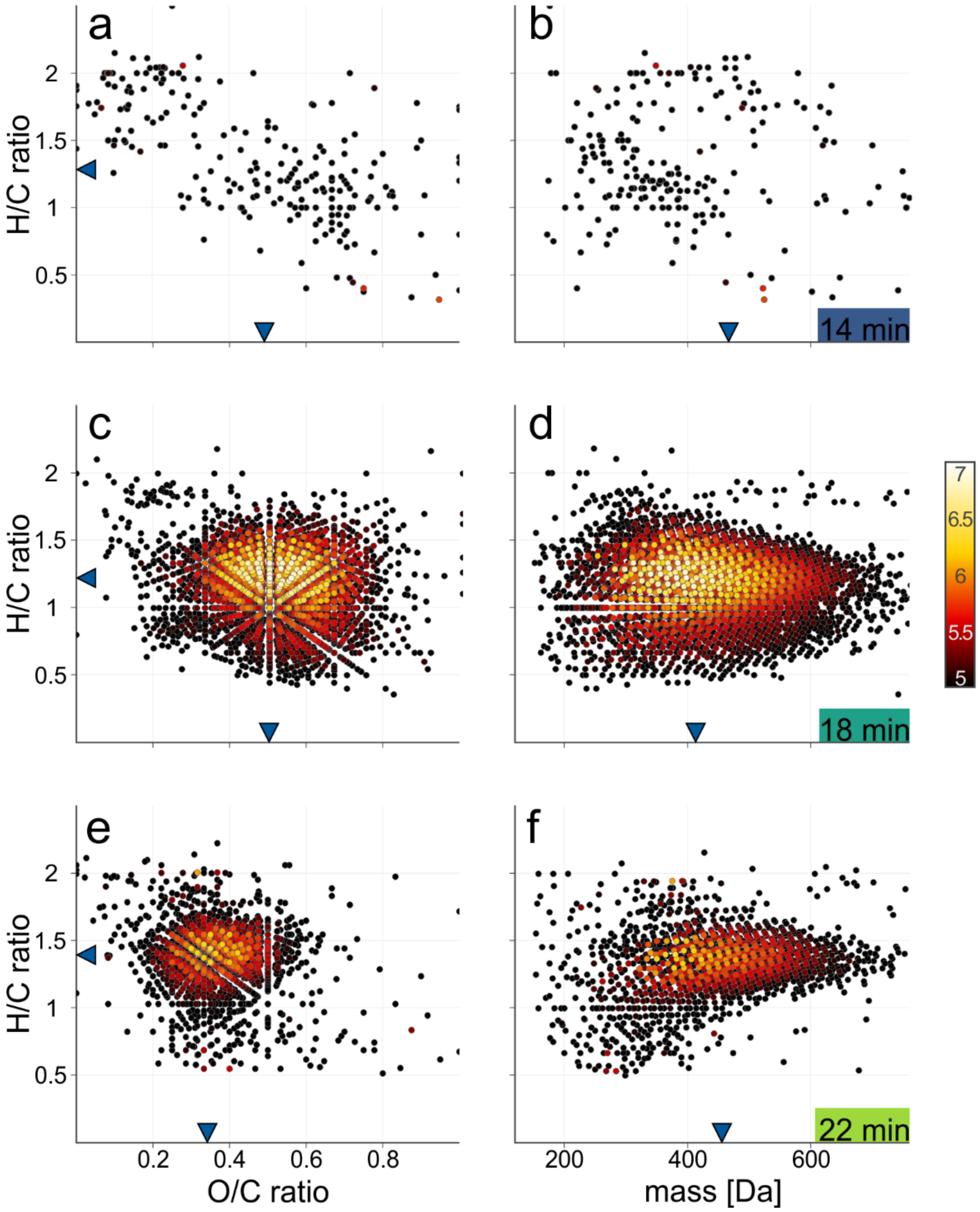

Figure SI 11: DOM chemodiversity in sample AO_{high}^{SPE}. Molecular H/C vs. O/C (a, c, e) and H/C vs. mass (b, d, f) for all detected MFs in selected segments at 14 min (a, b), 18 min (c, d) and 22 min (e, f), color coded by absolute intensity (log_{10}). The respective weighted average values are indicated by blue markers on the axes.

Marine and terrestrial marker in LC-FT-ICR MS

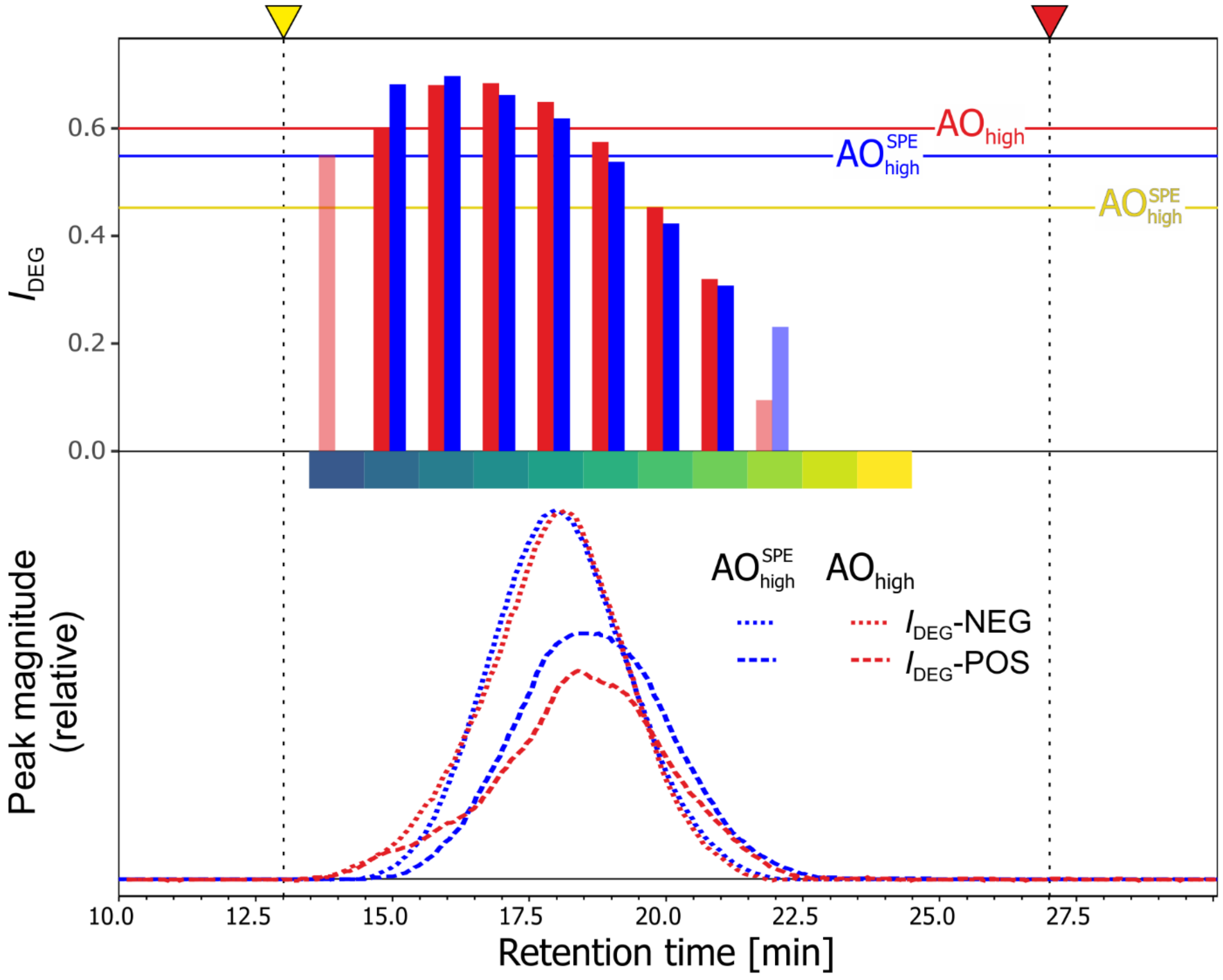

Figure SI 12: Evaluation of molecular formulas (MF) contained in the degradation index (I_{DEG}; Flerus et al. (2012). (top) Segment-wise I_{DEG} values for sample AO_{high} (red) and AO_{high}^{SPE} (blue) measured with LC-FT-ICR MS. The mean I_{DEG} values for the pseudo-DI spectra and the AO_{high}^{SPE} sample measured by DI-FT-ICR MS (yellow) indicated as lines. Segments, in which at least one I_{DEG}-MF was not detected are shown with transparent bars. (bottom) Extracted ion chromatograms (EIC) for the five I_{DEG}-NEG (dotted lines) and five I_{DEG}-POS (dashed lines) formulas in the original Arctic Ocean sample AO_{high} (red) and its SPE extract AO_{high}^{SPE} (blue). The EICs are scaled so that the maximum intensity in one sample (here: the apex of I_{DEG}-NEG EIC) is the same for both samples. The yellow and red marker and dashed line indicate the first (13 min) and 100% methanol (27 min) of the gradient.

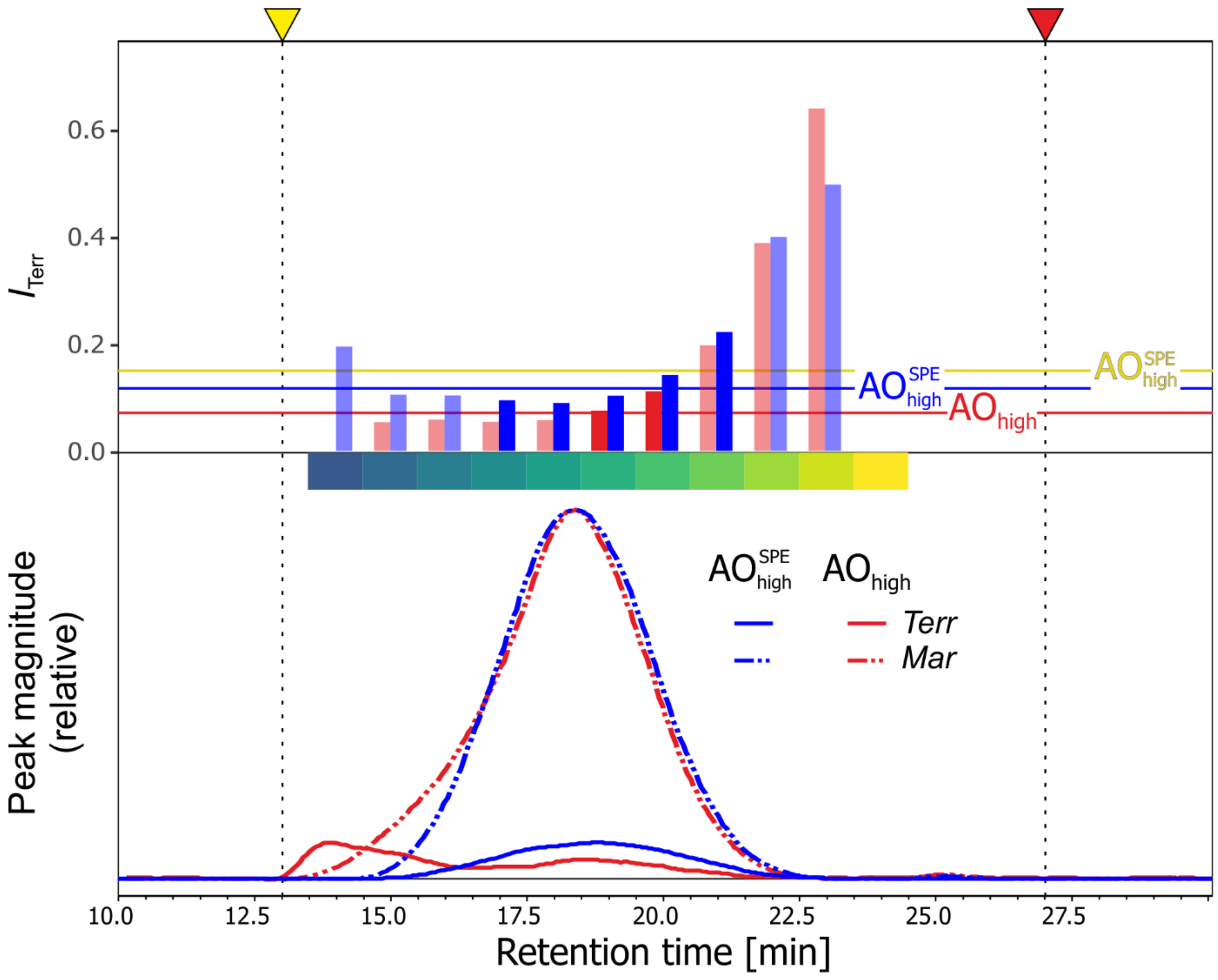

Figure SI 13: Evaluation of molecular formulas (MF) contained in the Terrestrial index (I_{Terr}; Medeiros et al. (2016). (top) Segment-wise I_{Terr} values for sample AO_{high} (red) and AO_{high}^{SPE} (blue) measured with LC-FT-ICR MS. The mean I_{Terr} values for the pseudo-DI spectra as well as the AO_{high}^{SPE} sample measured by DI-FT-ICR MS (yellow) is indicated as lines. Segments, in which more than 10 I_{Terr}-MF were not detected are shown with transparent bars. (bottom) Extracted ion chromatograms (EIC) for the 40 *Terr*-MF (solid lines) and 40 *Mar*-MF (dot-dashed lines) in the original Arctic Ocean sample AO_{high} (red) and its SPE extract AO_{high}^{SPE} (blue). The EICs are scaled so that the maximum intensity in one sample (here: the apex of *MAR* EIC) is the same for both samples. The yellow and red marker and dashed line indicate the first (13 min) and 100% methanol (27 min) of the gradient.

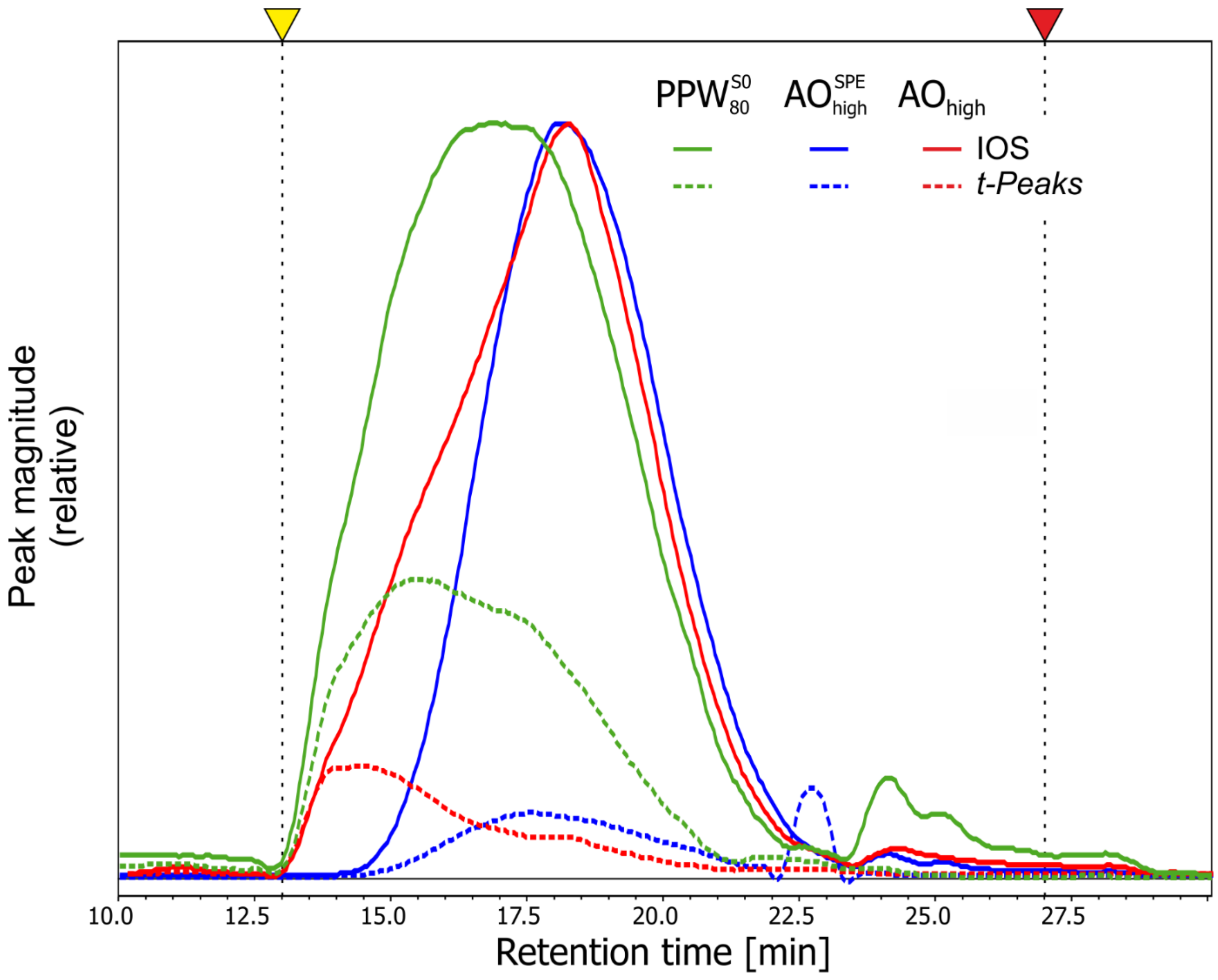

Figure SI 14: Island of stability (IOS)[4] and indicators of riverine input (t-Peaks).[5] Extracted ion chromatograms (EIC) for the 361 IOS-MF (solid lines) and 184 t-Peaks-MF (dotted lines) in samples AO$_{high}$ (red) and AO$_{high}^{SPE}$ (blue) and a peat-pore water (PPW$_{80}^{S0}$, green) measured with LC-FT-ICR MS. The EICs are scaled so that the maximum intensity in one sample (here: the apex of IOS EIC) is the same for both samples. The yellow and red marker and dashed line indicate the first (13 min) and 100% methanol (27 min) of the gradient.

Salt separation on the LC column

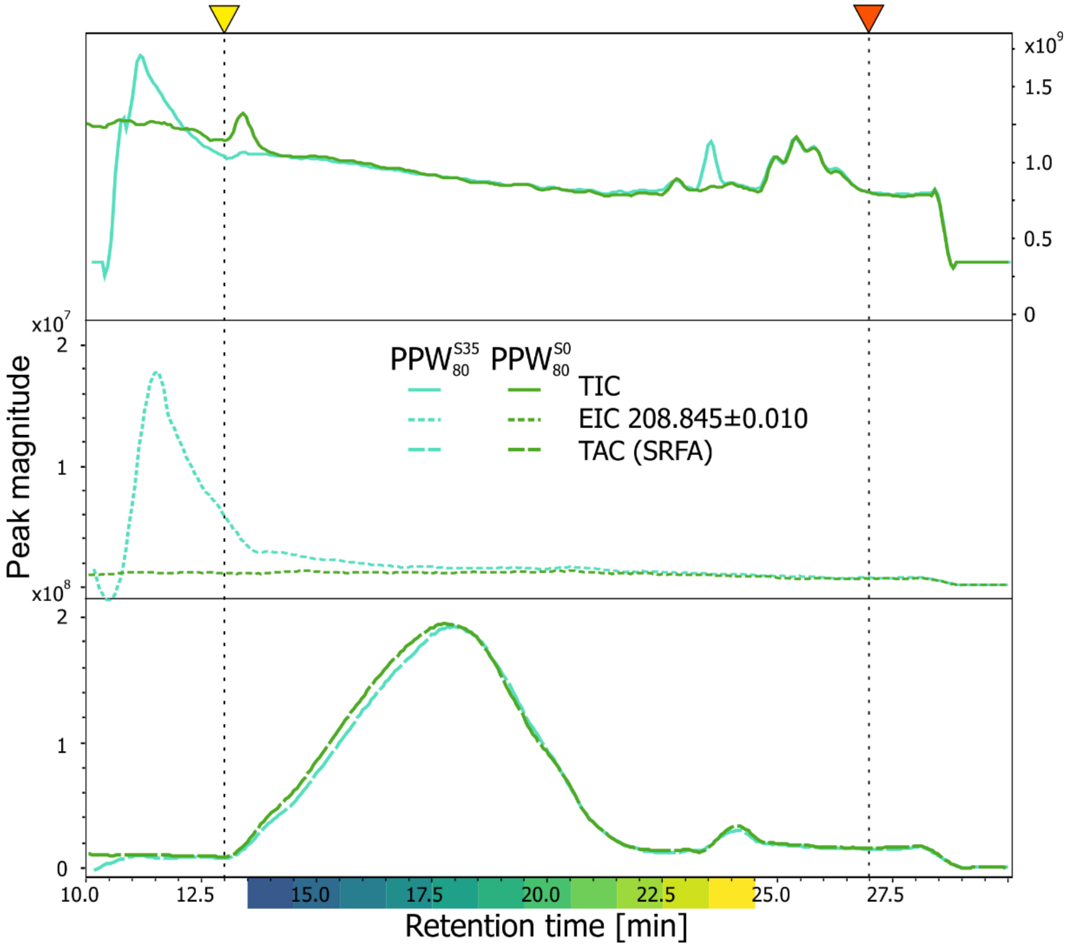

Figure SI 15: Separation of salt and DOM on the LC column. Total ion chromatograms (TIC, solid lines, top), extracted ion chromatograms (EIC, dotted lines, middle) using the m/z value of the prominent salt cluster $[Cl_4Na_3]^-$ (cf. Figure 1 in the main manuscript), and total assigned ion chromatograms (TAC, dashed lines, bottom) for a peat pore water without (PPW_{80}^{S0}: 80 µmol DOC L^{-1}) and with salt amendment (PPW_{80}^{S35}: 35 g NaCl L^{-1}). The yellow and red marker and dashed line indicate the first (13 min) and 100% methanol (27 min) of the gradient. The color bars indicate the LC retention time segments considered in this study. Note that not the full salt elution is visible from the EIC in the middle panel, since the valve directing the flow to the MS, was only switched at 10.5 min (cf. Figure SI 2) in the case of PPW_{80}^{S35}.

Model Compounds Intermediate Precision

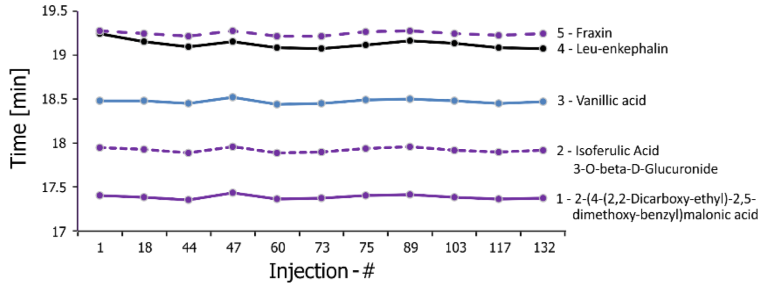

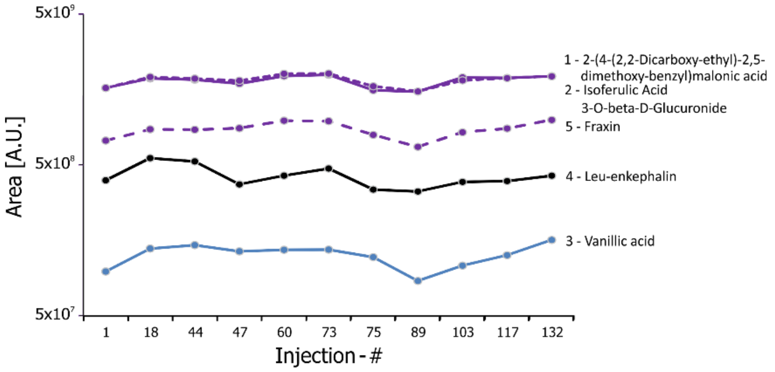

Figure SI 16: Intermediate precision of model compound peaks. Retention time (top) and peak area (bottom) for model compounds in SRFA (10 mg L^{-1}) injected within a sequence of 130 seawater samples. The numbers refer to the model compounds in Table SI 3.

Matrix Effects

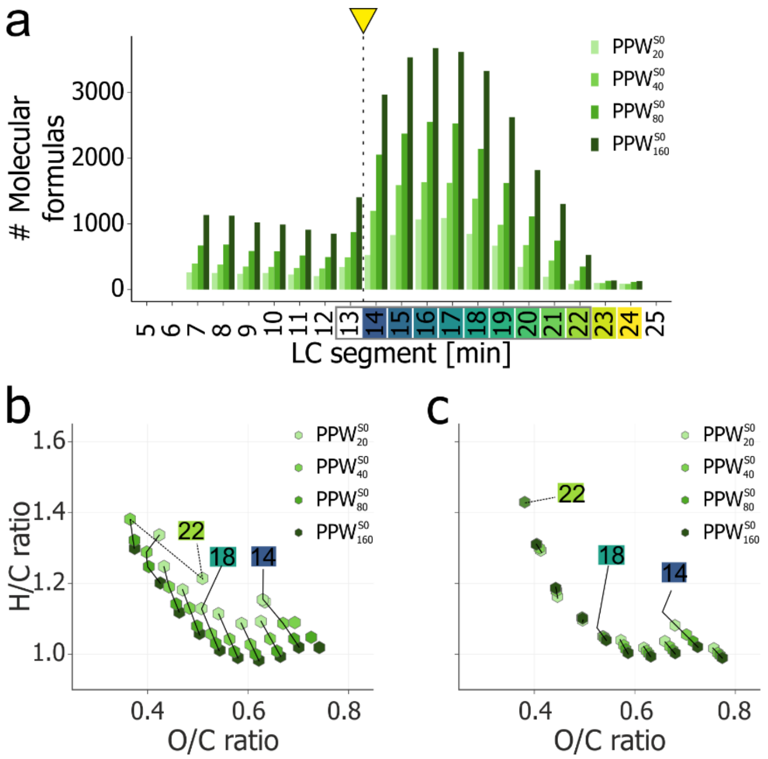

Figure SI 17: Effect of concentration on detectable PPW composition (without salt). (a) Number of molecular formulas (MFs) in each segment (= molecular features) for the concentration series (20 – 160 µmol DOC L^{-1}) of sample PPW^{S0}. Intensity-weighted average molecular H/C and O/C ratios for all segments ≥ 13 min for all concentration levels (20, 40, 80, and 160 µmol DOC L^{-1}) based on (b) all detected molecular formulas (20 µmol DOC L^{-1}: 86 < n < 1088; 160 µmol DOC L^{-1}: 528 < n < 3,677) in each segment and (c) only the segment-wise commonly detected MFs in all four concentration levels (1 < n < 814). The yellow marker and dashed line indicate the retention time at which the first methanol reaches the MS (13 min).

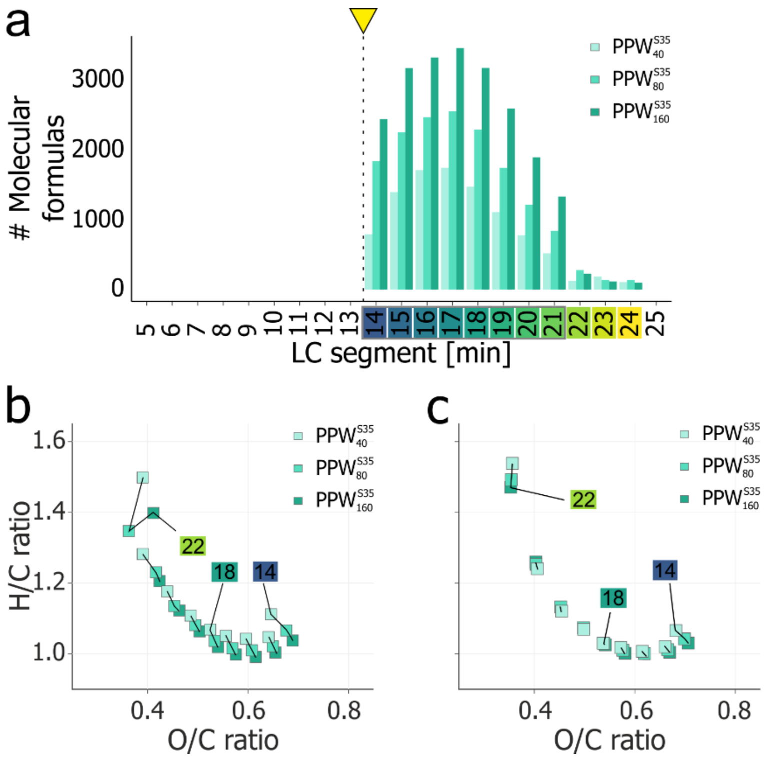

Figure SI 18: Effect of concentration on detectable PPW composition (with salt). (a) Number of molecular formulas (MFs) in each segment (= molecular features) for the concentration series (40 - 160 µmol DOC L^{-1}) of sample PPW^{S35}. Intensity-weighted average molecular H/C and O/C ratios for all segments ≥ 13 min for all concentration levels (40, 80, and 160 µmol DOC L^{-1}) based on (b) all detected molecular formulas (40 µmol DOC L^{-1}: 124 < *n* < 1,727; 160 µmol DOC L^{-1}: 226 < *n* < 3,422) in each segment and (c) only the segment-wise commonly detected MFs in all three concentration levels (2 < *n* < 1,389). The yellow marker and dashed line indicate the retention time at which the first methanol reaches the MS (13 min).

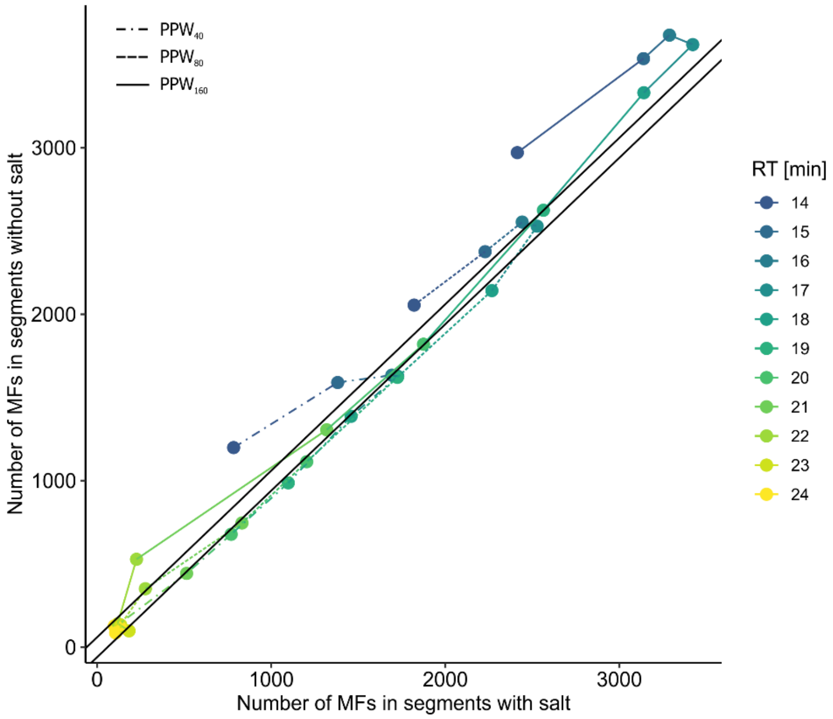

Figure SI 19: Salt matrix effects on the number of detected molecular formulas. Comparison of the number of molecular formulas (MFs) in individual LC segments (14 – 24 min) of samples PPW^{S0} and PPW^{S35} for three concentration levels (40 – 160 µmol DOC L^{-1}). Segment retention time is indicated by color, and the solid black lines indicate number of MFs variability as determined from sample AO_{low} (Figure SI 25). RT refers to the respective segment mean retention time.

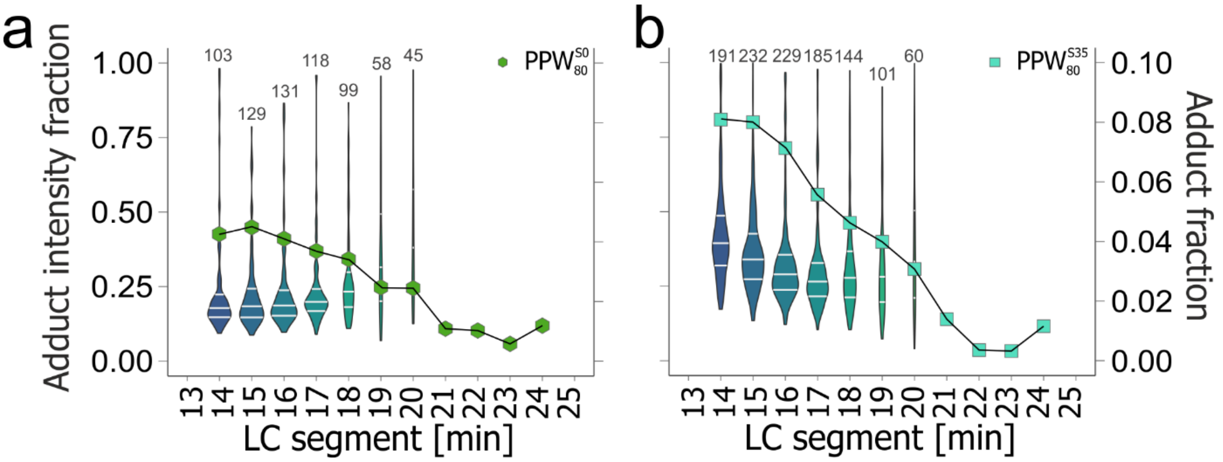

Figure SI 20: Potential Na-adducts in ESI(-)-LC-FT-ICR MS. Reassignment of molecular formulas in peat pore water samples PPW_{80}^{S0} and PPW_{80}^{S35} (with 80 µmol DOC L^{-1}) considering Na as potential element. Tentative Na-adducts ($[M\text{-}2H^{+}+Na^{+}]^{-}$) for highly polar DOM molecules were identified by linking a MF ($C_cH_hN_nO_oS_s$) to its potential Na-adduct ($C_cH_{h-1}Na_1N_nO_oS_s$). The number of potential Na-adducts detected in this way is shown as fraction of total number of MF (right y-axis: "Adduct fraction"; lines) in each segment. Up to 8% of MF can potentially have a Na-adduct in the most polar DOM segment (14 min) of the salt-amended PPW_{80}^{S35} sample, while this number is 4 % for the salt-free PPW_{80}^{S0} sample. Likewise, the intensity for each potential Na-adduct is calculated as fraction of the assigned intensity for the Na-adduct MF to the summed intensity for the Na-adduct MF and its protonated counterpart MF: Intensity($[M\text{-}2H^{+}+Na^{+}]^{-}$)/(Intensity($[M\text{-}2H^{+}+Na^{+}]^{-}$) + Intensity($[M\text{-}H^{+}]^{-}$)). The distribution of these intensity fractions for each segment is shown (left y-axis: "Adduct intensity fraction"; violins). Only for the tentative Na-adducts, the mean intensity fraction is ~40% in the most polar DOM segment (14 min) of the salt-amended PPW_{80}^{S35} sample, while this number is ~25 % for the salt-free PPW_{80}^{S0} sample. The number above the violins represent the number of potential Na-adducts in each segment.

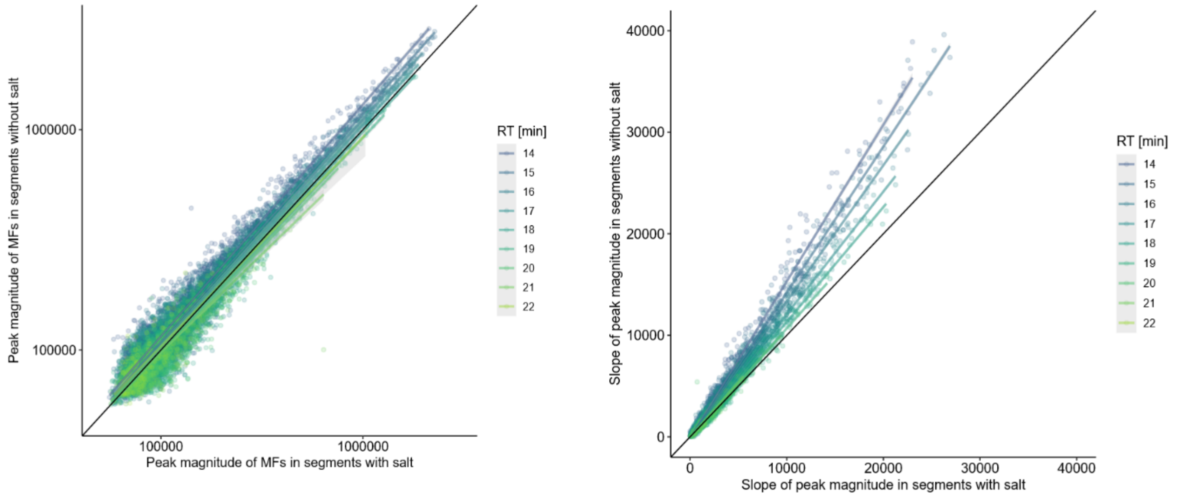

Figure SI 21: Salt matrix effects on raw peak intensity: (a) Comparison of the raw peak intensity of assigned molecular formulas (MFs) in individual LC segments (14 – 24 min) of samples PPW^{S0} and PPW^{S35} injected at 80 µmol DOC L^{-1}. (b) Calculated slopes for the regression of raw peak magnitude with injected DOC concentration (40 – 160 µmol DOC L^{-1}). Mean segment retention time is indicated by color and segment-wise linear regression is displayed.

Detector Response and Linearity

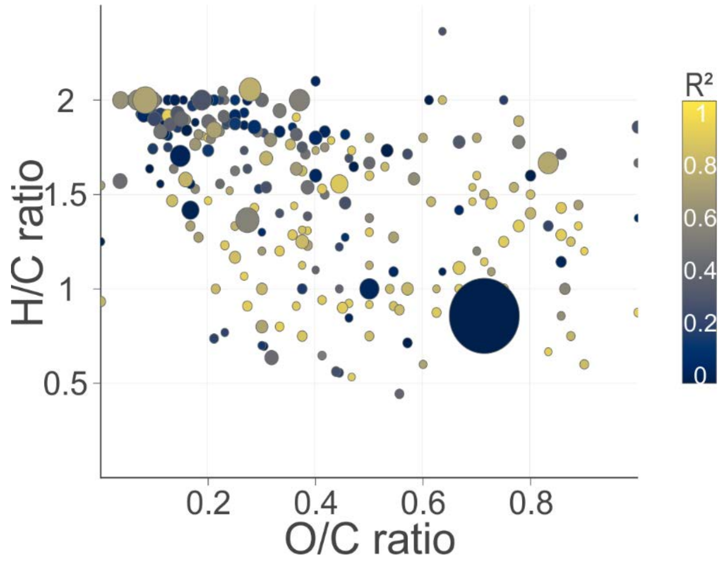

Figure SI 22: Pearson's regression coefficient (R^2) for raw mass peak intensities <u>without</u> significant correlation to DOC concentration. Molecular formulas (MFs) shown were detected in segments of the PPW^{S0} samples at all concentrations (20 - 160 µmol DOC L^{-1}). MF with R^2 values < 0.90 (p > 0.05) are shown (n = 262), representing 4% (19 min) – 19% (21 min) of MFs that were detected at all concentrations (cf. Figure 4b in main text). The size of the dots represents the signal-to-noise ratio (S/N) of the respective MF in the 20 µmol DOC L^{-1} sample (smallest: S/N = 4, largest: S/N = 372). MFs with high H/C and low O/C ratio are typically CHO compounds and elute with the first MeOH in the gradient and may be related to contaminants from the LC system.

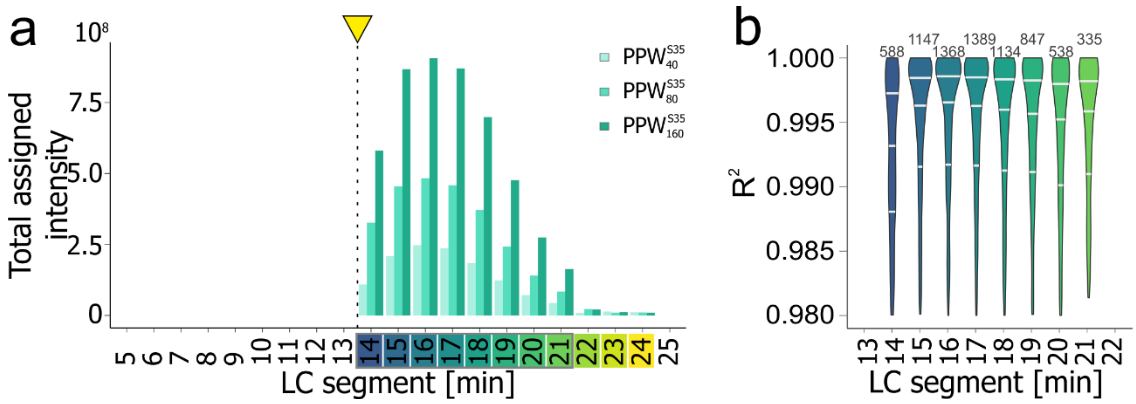

Figure SI 23: Linearity of ICR detector response for PPW with salt. (a) Summed intensity of assigned molecular formulas (total assigned intensity) in individual LC-segments (14-24 min, 11 segments) of an original peat pore water (PPW^{S35}) with constant 35 g L^{-1} salt concentration. Colors on the retention time axis indicate data displayed in (b). (b) Linear regression of raw mass peak intensities for molecular formulas (MFs) detected in segments of PPW^{S35}. R^2 values > 0.98 (corresponding to a significant linear regression at α = 0.01) are displayed for those MFs that were detected at all concentrations (335 < n < 1,389). Segments < 14 min and > 21 min were omitted due to the low number of detected MFs for the 40 µmol DOC L^{-1}. The yellow marker and dashed line indicate the retention time at which the first methanol reaches the MS (13 min).

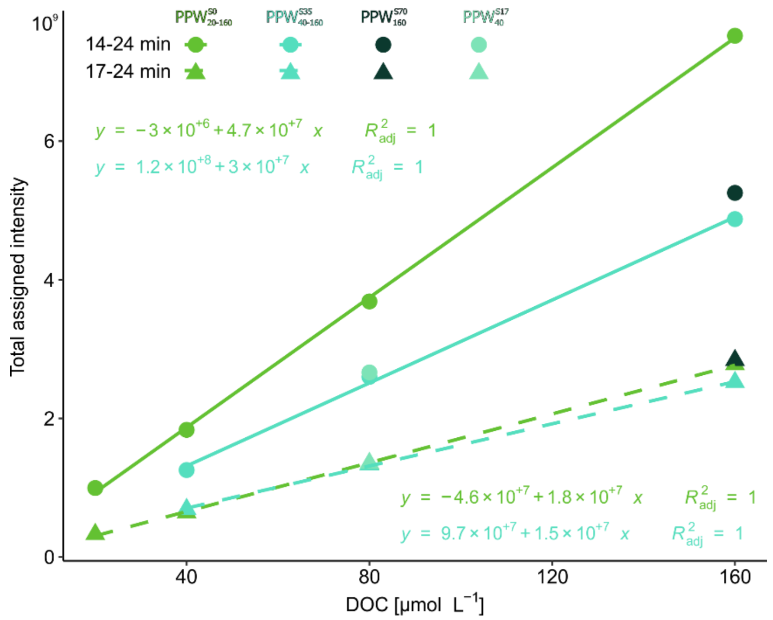

Figure SI 24: Comparison of ICR detector response with and without salt addition. Total assigned intensities (TAI) in samples PPW^{S0} (green; 20 - 160 µmol DOC L^{-1}) and PPW^{S35} (light blue; 40 - 160 µmol DOC L^{-1}). Values for PPW^{S17} (80 µmol DOC L^{-1}) and PPW^{S70} (160 µmol DOC L^{-1}) were added to simulate estuarine and sea ice brine samples. The data represent TAI for all detectable segments (circles; 14 - 24 min) and those segments that were not affected by salt suppression (triangles; 17 - 24 min). Respective regression equations are shown with the respective colors of PPW^{S0} (green) and PPW^{S35} (light blue).

Repeatability

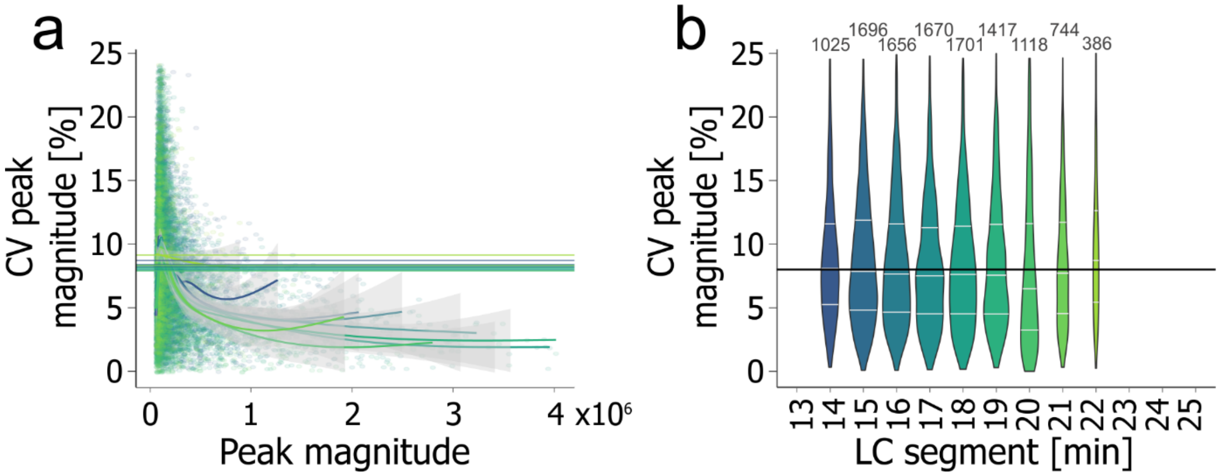

Figure SI 25: Repeatability of raw peak magnitudes in sample AO_{low}. (a) Peak intensity variation (calculated as coefficient of variation (CV) from raw peak magnitudes) across triplicate injections of sample AO_{low} (55 µmol DOC L^{-1}) as a function of peak magnitude. Segment-wise local polynomial smoothing and mean CV (8.3 – 9.6%) are indicated according to the colors in (b). (b) Distribution of CV values (with 25th, median and 75th percentile as white lines) of molecular formulas (MFs) for individual LC segments (14 – 22 min, 386 < *n* < 1,701, median CV: 6.4 – 8.7%). The solid black line in (b) indicates the overall mean CV of peak magnitude.

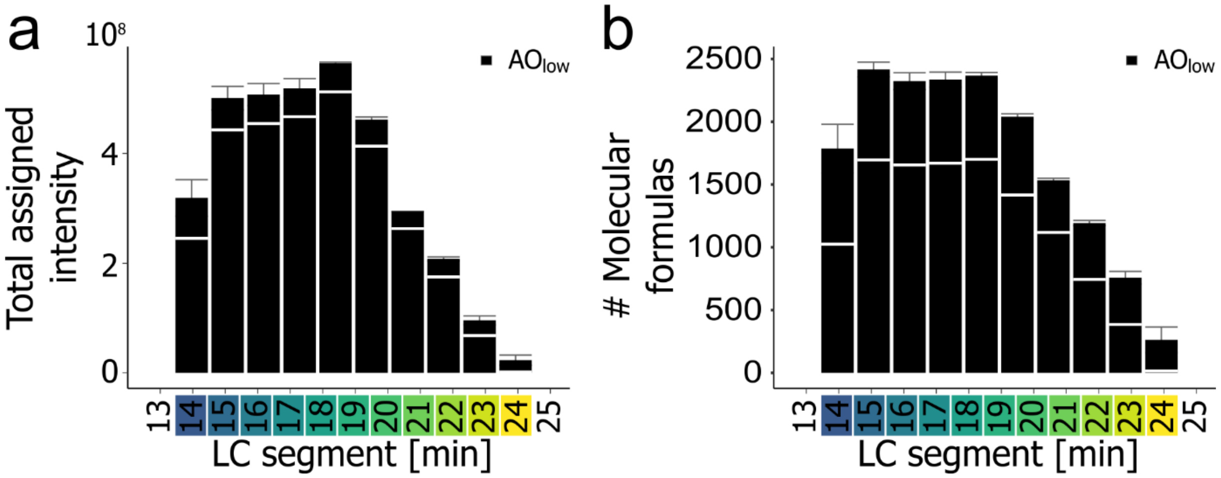

Figure SI 26: Repeatability of (a) total assigned intensity (summed intensity of peaks having a formula assignment) and (b) number of molecular formulas (MFs) in individual LC-segments (14 – 24 min, 11 segments) for an original seawater sample (AO_{low}; 55 µmol DOC L^{-1}). Segment-wise variability is indicated by error bars and the corresponding values for all shared MFs (386 < *n* < 1,701) across triplicates within each segment is indicated by the white lines in each bar.

Sample pH Effect

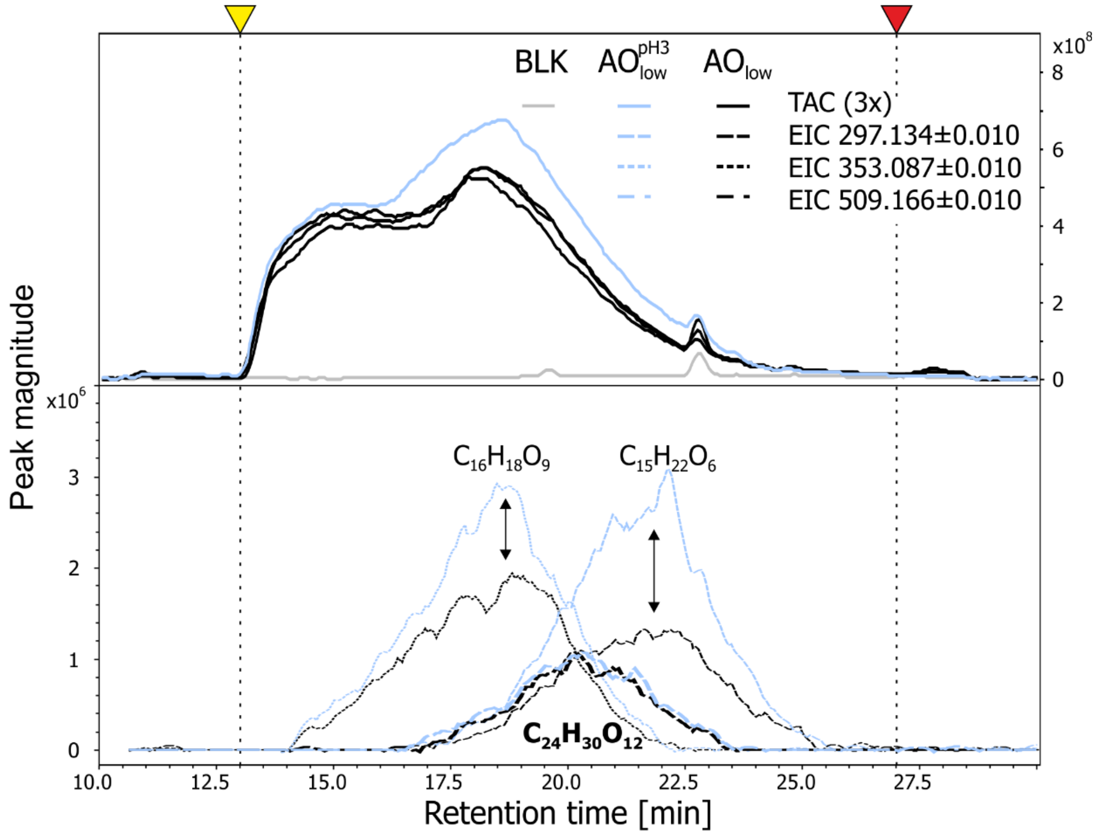

Figure SI 27: Injection pH effect. Total assigned ion chromatograms (TAC, solid lines, right y-axis) and extracted ion chromatograms (EIC, dashed lines, left y-axis) for the Arctic Ocean sample AO_{low} injected at native pH (black, measured as triplicates) and at pH 3 (AO_{low}^{pH3}: light blue). Three m/z values were selected, representing a low (m/z 297.134: $C_{15}H_{22}O_6$), medium (m/z 353.087: $C_{16}H_{18}O_9$) and high mass DOM compound (m/z 509.166: $C_{24}H_{30}O_{12}$). The yellow and red marker and dashed line indicate the first (13 min) and 100% methanol (27 min) of the gradient.

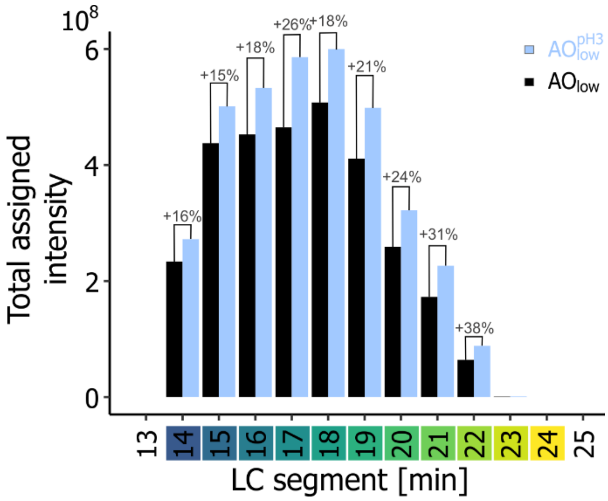

Figure SI 28: Effect of sample pH during injection. Summed intensity of assigned molecular formulas (total assigned intensity) in sample AO_{low} injected at native concentration (55 µmol DOC L^{-1}) and pH (black bars) and with pH adjusted to 3 by formic acid (blue bars) before injection (AO_{low}^{pH3}). The percent difference is indicated for each LC segment.

Original Water vs. SPE

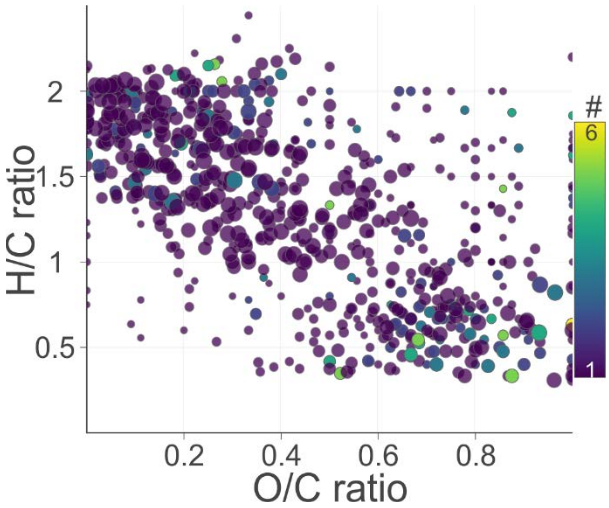

Figure SI 29: Unique molecular formulas (MFs) detected in early eluting polar DOM (LC segments 7 – 13 min, n = 711) in sample AO_{high}^{SPE}, color coded by their frequency of occurrence ($1 \le n \le 6$). The cluster of MFs in the top left corner represents large, nonpolar molecules unlikely to elute with the most polar DOM fraction (cf. Jennings et al. (2022)[6]) which may be related to LC-system derived contaminants. These MF are excluded from Figure SI 30 and Figure SI 31.

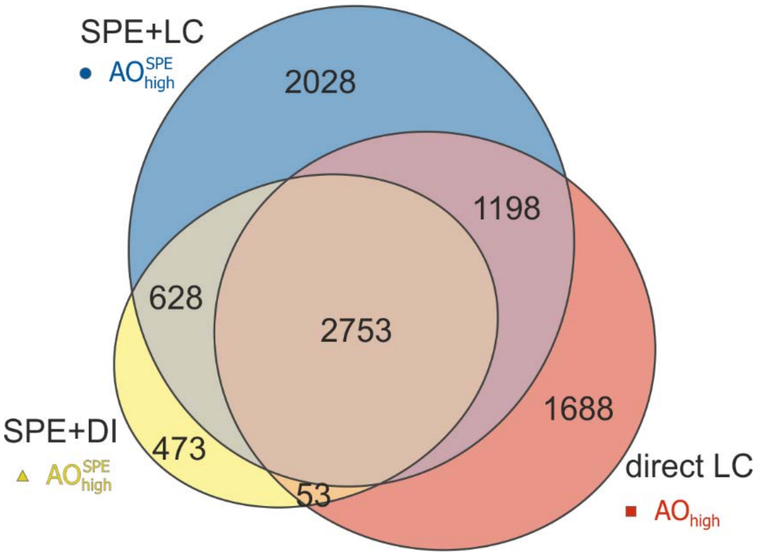

Figure SI 30: Comparison of original water and extract analyzed by direct-infusion (DI) and LC-FT-ICR MS. Venn diagram for molecular formulas (MFs) detected at S/N > 4 for an original (AO_{high}, red) and a PPL-extracted sample (AO_{high}^{SPE}, blue), both measured at 88 µmol DOC L^{-1} with LC-FT-ICR MS, and a sample measured with DI at 0.8 mmol DOC L^{-1} (AO_{high}^{SPE}, yellow). In contrast to Figure SI 29, only LC segments > 14 min are used.

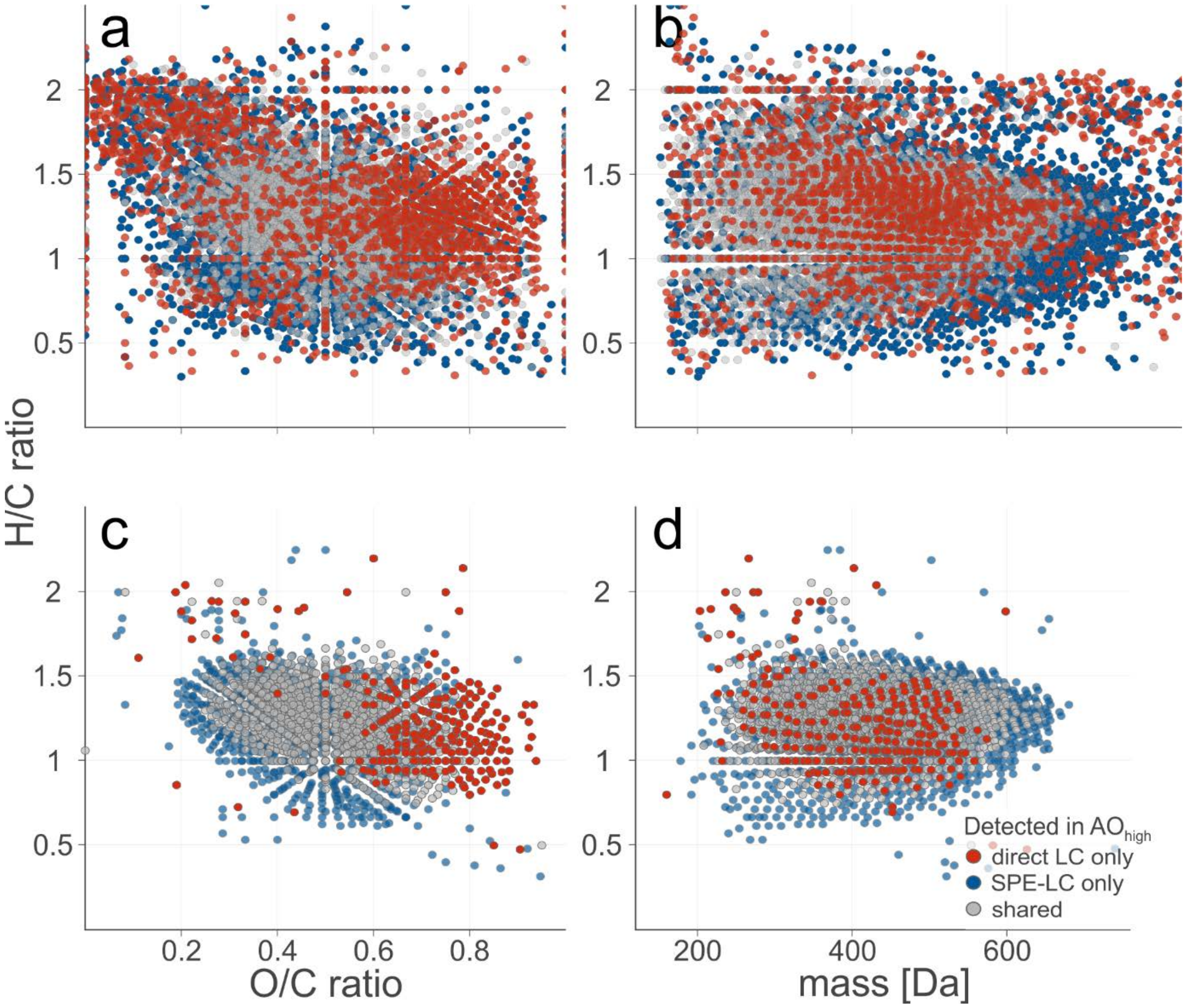

Figure SI 31: Comparison of original water and extract analyzed by LC-FT-ICR MS. Molecular formulas (MFs) detected for an original (AO_{high}) and PPL-extracted sample (AO_{high}^{SPE}), both measured at 88 µmol DOC L^{-1} with LC-FT-ICR MS. MFs with an S/N ratio > 4 (a, b) and > 15 (c, d) are displayed. Molecular H/C vs. O/C (a, c) and H/C vs. mass (b, d) for all detected MFs shared (gray, *n* = 3,951/1,266) or uniquely detected in original (AO_{high}, red, *n* = 1,741/279), or the PPL extract (AO_{high}^{SPE}, blue, *n* = 2,656/785). Only LC segments > 14 min are used.

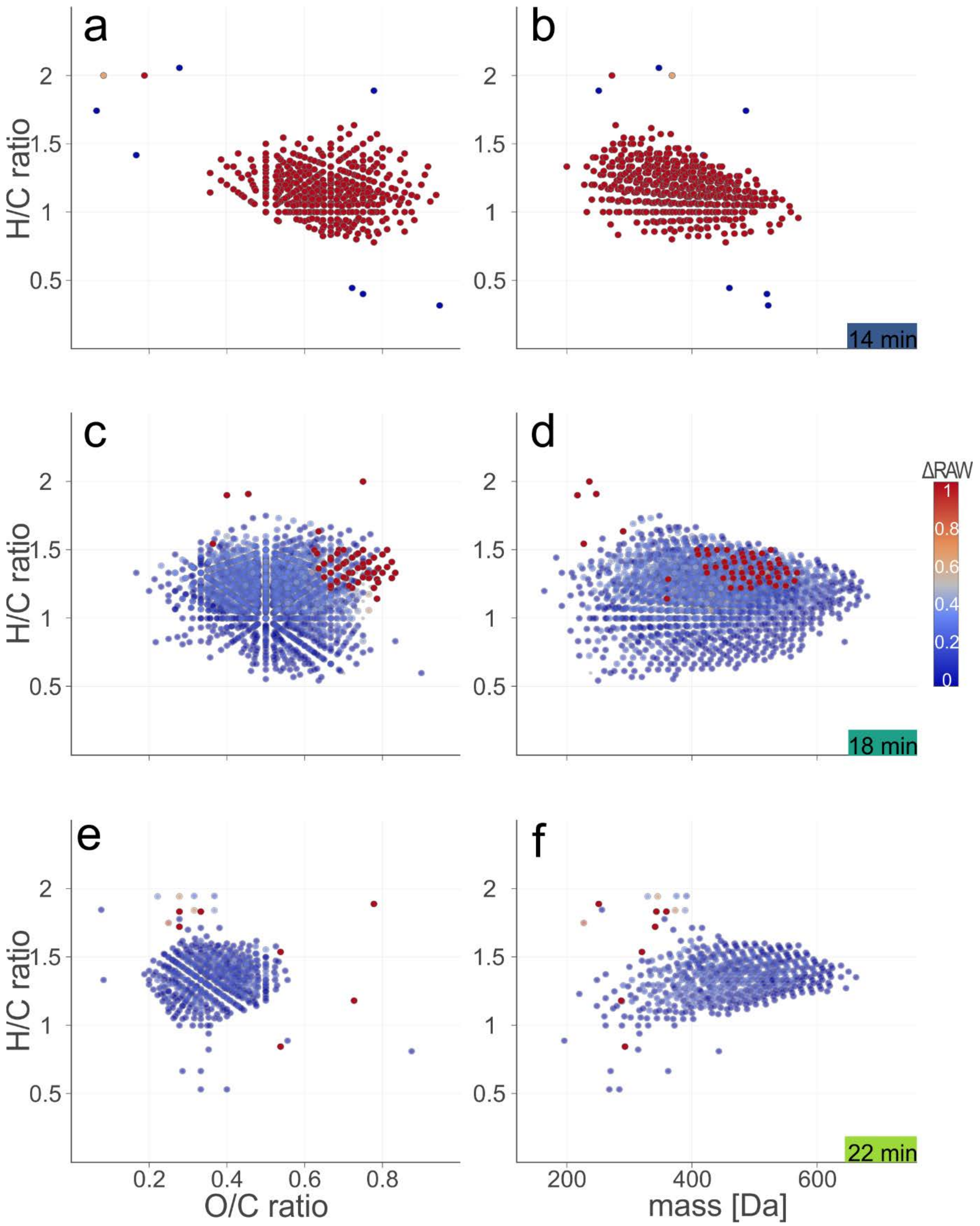

Figure SI 32: Comparison of detected molecular formulas in sample AO_{high} and AO_{high}^{SPE}. Molecular H/C vs. O/C (a, c, e) and H/C vs. mass (b, d, f) for all detected, shared molecular formulas (MFs) in the original seawater sample AO_{high} and its SPE extract AO_{high}^{SPE} for selected segments at 14 min (a, b), 18 min (c, d) and 22 min (e, f) and color coded by the normalized raw peak magnitude difference ($\Delta RAW = RAW[AO_{high}]/(RAW[AO_{high}^{SPE}])+RAW[AO_{high}]$). Red colors indicate higher intensity in the original seawater sample AO_{high}, blue colors in the PPL extracted sample AO_{high}^{SPE}. Note that the color scale definition deviates from the RAW scale used in the main text.

Original Water vs. DI

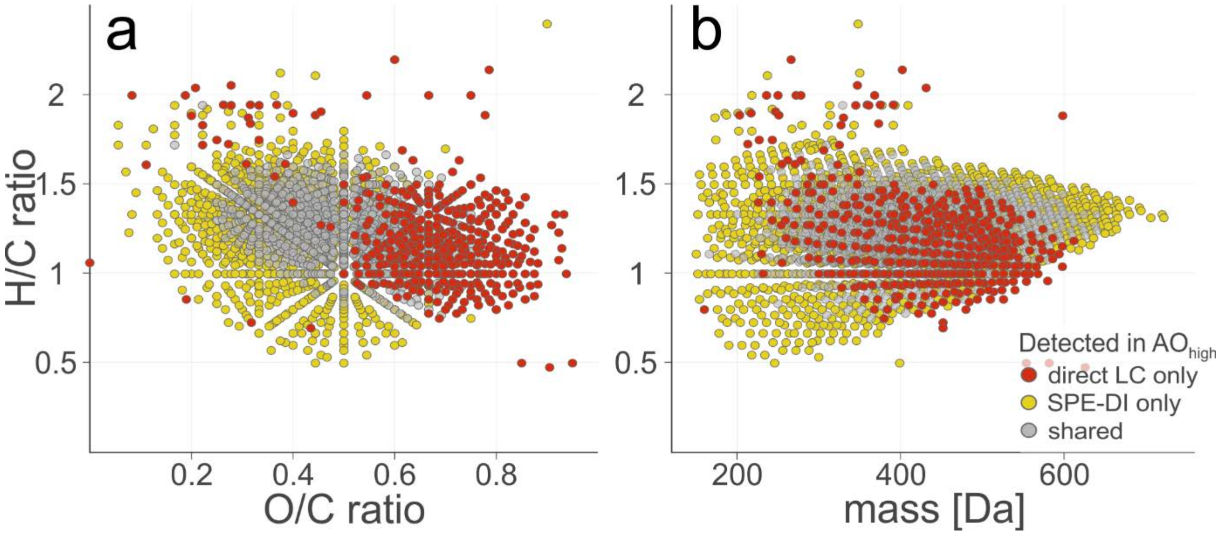

Figure SI 33: Comparison of direct infusion and LC analysis. Molecular formulas (MFs) detected by direct LC measurement of an original sample (AO_{high}) measured at 88 µmol DOC L^{-1} with LC-FT-ICR MS and its PPL-extract (AO_{high}^{SPE}) measured at 0.8 mmol DOC L^{-1} with direct infusion (DI). Molecular H/C vs. O/C (a) and H/C vs. mass (b) for all detected MFs shared (gray, *n* = 1,067) or uniquely detected in original (AO_{high}, red, *n* = 478), or the PPL extract measured with DI (AO_{high}^{SPE}, yellow, *n* = 947). Only MFs with an S/N ratio > 15 are displayed. For a comparison with the PPL-extracted sample (AO_{high}^{SPE}) measured with LC-FT-ICR MS cf. Figure SI 31.

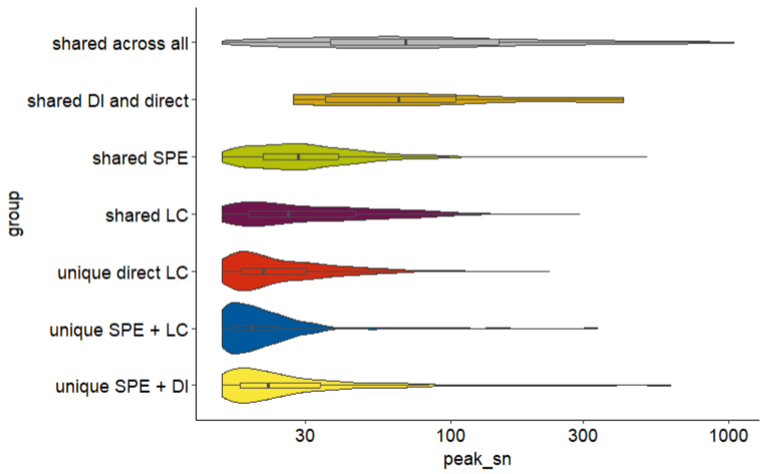

Figure SI 34: Shared and uniquely detected molecular formulas in sample AO_{high}. Distribution of peak S/N values for all molecular formulas (MFs) detected in an original seawater sample AO_{high} and its SPE extract AO_{high}^{SPE} (both injected at 88 µmol DOC L^{-1}) with LC-FT-ICR-MS. The SPE extract AO_{high}^{SPE} was also measured via direct infusion (DI) FT-ICR-MS (injected at 0.8 mmol DOC L^{-1}).

Additional references

(1) Han, L.; Kaesler, J.; Peng, C.; Reemtsma, T.; Lechtenfeld, O. J., Online Counter Gradient LC-FT-ICR-MS Enables Detection of Highly Polar Natural Organic Matter Fractions. *Anal. Chem.* **2021,** *93* (3), 1740-1748.
(2) Patriarca, C.; Bergquist, J.; Sjöberg, P. J. R.; Tranvik, L.; Hawkes, J. A., Online HPLC-ESI-HRMS Method for the Analysis and Comparison of Different Dissolved Organic Matter Samples. *Environ. Sci. Technol.* **2017**.
(3) Flerus, R.; Lechtenfeld, O. J.; Koch, B. P.; McCallister, S. L.; Schmitt-Kopplin, P.; Benner, R.; Kaiser, K.; Kattner, G., A molecular perspective on the ageing of marine dissolved organic matter. *Biogeosciences* **2012,** *9* (6), 1935-1955.
(4) Lechtenfeld, O. J.; Kattner, G.; Flerus, R.; McCallister, S. L.; Schmitt-Kopplin, P.; Koch, B. P., Molecular transformation and degradation of refractory dissolved organic matter in the Atlantic and Southern Ocean. *Geochim. Cosmochim. Acta* **2014,** *126*, 321-337.
(5) Medeiros, P. M.; Seidel, M.; Niggemann, J.; Spencer, R. G. M.; Hernes, P. J.; Yager, P. L.; Miller, W. L.; Dittmar, T.; Hansell, D. A., A novel molecular approach for tracing terrigenous dissolved organic matter into the deep ocean. *Global Biogeochem. Cycles* **2016,** *30* (5), 689-699.
(6) Jennings, E.; Kremser, A.; Han, L.; Reemtsma, T.; Lechtenfeld, O. J., Discovery of Polar Ozonation Byproducts via Direct Injection of Effluent Organic Matter with Online LC-FT-ICR-MS. *Environ. Sci. Technol.* **2022,** *56* (3), 1894-1904.
